# USP7- and PRMT5-dependent G3BP2 stabilization drives de novo lipogenesis and tumorigenesis of HNSC

**DOI:** 10.1038/s41419-023-05706-2

**Published:** 2023-03-06

**Authors:** Nan Wang, Tianzi Li, Wanyu Liu, Jinhua Lin, Ke Zhang, Zhenhao Li, Yanfei Huang, Yufei Shi, Meilan Xu, Xuekui Liu

**Affiliations:** 1 Laboratory of Cell and Molecular Biology, School of life sciences, Jiaying University, Meizhou, China; 2 State Key Laboratory of Oncology in South China, Collaborative Innovation Center for Cancer Medicine, Sun Yat-sen University Cancer Center, Guangzhou, China

**Keywords:** Post-translational modifications, Cancer metabolism

## Abstract

GTPase-activating protein-binding protein 2 (G3BP2) is a key stress granule-associated RNA-binding protein responsible for the formation of stress granules (SGs). Hyperactivation of G3BP2 is associated with various pathological conditions, especially cancers. Emerging evidence indicates that post-translational modifications (PTMs) play critical roles in gene transcription, integrate metabolism and immune surveillance. However, how PTMs directly regulate G3BP2 activity is lacking. Here, our analyses identify a novel mechanism that PRMT5-mediated G3BP2-R468me2 enhances the binding to deubiquitinase USP7, which ensures the deubiquitination and stabilization of G3BP2. Mechanistically, USP7- and PRMT5-dependent G3BP2 stabilization consequently guarantee robust *ACLY* activation, which thereby stimulating de novo lipogenesis and tumorigenesis. More importantly, USP7-induced G3BP2 deubiquitination is attenuated by PRMT5 depletion or inhibition. PRMT5-activity dependent methylation of G3BP2 is required for its deubiquitination and stabilization by USP7. Consistently, G3BP2, PRMT5 and G3BP2 R468me2 protein levels were found positively correlated in clinical patients and associated with poor prognosis. Altogether, these data suggest that PRMT5-USP7-G3BP2 regulatory axis serves as a lipid metabolism reprogramming mechanism in tumorigenesis, and unveil a promising therapeutic target in the metabolic treatment of head and neck squamous carcinoma.

## Introduction

In addition to genetic and epigenetics alterations, aberrant lipid metabolism in cancer is becoming increasingly recognized as a hallmarker [1, 2]. Accumulating evidence revealed that hyperactive fatty-acid synthesis, uptake and storage pathways lead to tumorigenesis. Hence, the inhibition of lipid metabolic pathways might be a potential therapeutic target in cancers. Nevertheless, the association between lipid metabolism and the pathological development of cancer is not well illustrated.

GTPase-activating protein-binding proteins 1 and 2 (G3BP1 and G3BP2) are originally recognized as core components that contributes to stress granules (SGs) assembly [3]. Several lines of study underline the importance of G3BP2 in regulating the formation of SGs, RNA metabolism and human diseases, including cardiac hypertrophy and diabetic nephropathy [4, 5]. Inhibition of G3BP2 prevents tumor growth and augments the accumulation of nuclear p53 in mice [6, 7]. G3BP2 is also required for the translocation of p53 and RanBP2-mediated p53 SUMOylation in prostate cancer cells [8]. In addition, G3BP2-MG53 complex participates in the formation of GSs, which regulates lung tumor progression [9]. To date, no known role for G3BP2 in lipogenesis and its molecular detail functions in specific processes by post-translational modification (PTM) have been previously identified. Much more need to be studied regarding the role of G3BP2 in tumorigenesis.

Protein arginine methyltransferase 5 (PRMT5), a dominant type II protein arginine methyltransferase, plays an important role by interacting with cytoplasmic and nuclear molecules, such as transcription factors, elongation factors and splicing factors [10]. Importantly, PRMT5 exerts oncogenic driver and its dysregulation has been linked to human diseases including malignancies [11–13]. Recent studies revealed that epigenetic modifications arise as important mechanisms involved in reprogramming of metabolic patterns. For instance, PRMT5-methylation of KLF4 and then retards its ubiquitination by pVHL and stabilizes KLF4, thereby enhancing oncogenic signaling in breast cancer [14, 15]. PRMT5-mediated SREBP1 methylation inhibits its phosphorylation by GSK3β, suggesting the PTMs role of PRMT5 in HCC [16]. Despite some studies have highlighted the importance of PRMT5 in cancers, the PTMs role of this enzyme in de novo lipogenesis needs further exploration. Therefore, understanding the regulatory relationship between epigenetic modifications and dysfunctional energy metabolism will aid to elucidate the mechanisms of cancer development.

In the current work, we show that PRMT5 is responsible for G3BP2 accumulation by inducing its binding and deubiquitination by Ubiquitin-Specific Peptidase 7 (USP7). USP7-mediated G3BP2 deubiquitination and stabilization are attenuated by PRMT5 depletion or chemical inhibition of methyl-transferase activity of PRMT5. PRMT5 and USP7 serve as a G3BP2-sensitive ‘switch’ regulating G3BP2 stabilization and lipid metabolism reprogramming, which leads to lipogenesis and aggressive malignancy of head and neck squamous carcinoma (HNSC). PRMT5-USP7-G3BP2 regulatory complex is an essential driver for tumorigenesis.

## Materials and methods

### Cell lines and transfections

Human embryonic kidney cells (HEK293) were purchased from the type culture collection of the Chinese Academy of Sciences (Shanghai, China). HNSC cells (Tu686, Tu212, CAL-27, SCC25, HSC3) were purchased from Guangzhou Juyan Biological Technology (Guangzhou, China). Cells were maintained in RPMI-1640 or in Dulbecco’s modified Eagle’s medium (DMEM, Gibco). All culture media were supplemented with 10% FBS and 1% Penicillin-Streptomycin in a 37 °C humidified incubator with 5% CO_2_. All of the cells were authenticated and no mycoplasma contamination was detected in the cell lines used in this study. Wild-type G3BP2, PRMT5, truncated and R468K-mutant G3BP2 plasmids were synthesized by GeneCopoeia (Rockville, MD, USA). Constructs, siRNAs, and shRNAs were transfected into HEK293 and HNSC cell lines in accordance with the instructions of Lipofectamine 3000 reagents, respectively (Life Technologies/Invitrogen). Supplementary Table 1 contains detailed information about the shRNA and siRNA sequences. The reagent used in this study: cycloheximide (CHX) 50 μg/mL, MG132 40 μM.

### Immunoprecipitation and Immunoblotting analysis

The indicated cells were lysed with RIPA buffer (10 Mm Tris-HCl, pH 7.4, 150 mM NaCl, 1 mM EDTA, and 0.5% Nonidet P-40) supplemented with protease inhibitor cocktail. After incubation on ice for 30 min, 30 μl protein A/G magnetic beads was used to preclear cell extracts, followed by incubation with either IgG (1:100, 3900 S, Cell Signaling Technology), USP7 primary antibody (1:100, GTX125894, GeneTex) or PRMT5 (1:50, 79998, Cell Signaling Technology) antibody overnight at 4 °C. The immune complexes were followed by incubation with 40 μl protein A/G beads for 2 h. Immunoprecipitates were washed and subjected to Western blot, silver staining and mass spectrometry analysis. Silver staining was performed using the Fast Silver Stain Kit (Beyotime) and mass spectrometry was carried out by Novogene, in Beijing, China. Immunoblotting assay was performed as previously described [17]. Antibodies specific to human proteins were anti-PRMT5 (ab109451, Abcam), anti-G3BP2 (16276-1-AP; Proteintech and ab86135; Abcam), anti-Flag (9A3; Cell Signaling Technology), anti-α-Tubulin (T6074, MilliporeSigma), anti-USP7 (4833 S, Cell Signaling Technology), anti-ACLY (4332, Cell Signaling Technology), anti-FASN (3180, Cell Signaling Technology), anti-PPARγ (33436 M, Bioss Antibodies), anti-SCD1 (3787 R, Bioss Antibodies), anti-nSREBP1 (ab159577, Abcam) anti-ELOVL6 (ab69857, Abcam), anti-ACSL3 (ab151959, Abcam) and anti-methy-G3BP2 was a custom-made polyclonal antibody.

### Luciferase reporter analysis

The *ACLY* and *FASN* promoters were prepared by PCR and inserted into pGL3-Basic vectors. HEK293 and PRMT5 (KO) cells were grown in DMEM and transiently co-transfected with plasmids containing 3’-UTR of wild-type (WT) or R468K (MUT) from G3BP2 and PRMT5, along with *ACLY* or *FASN* luciferase reporter plasmids using Lipofectamine 3000. Luciferase activities were measured 60 h after transfection using the dual luciferase reporter assay system kit (Promega, Madison, USA) and normalized to Renilla luciferase activity, each experiment was repeated in triplicate.

### CRISPR-Cas9 knockout cell line

To generate PRMT5-Tu686 knockout cell lines, the sgRNA sequences were cloned into LentiCRISPRv2 plasmid, then the recombinant viral plasmids and viral packaging plasmids (psPAX2 and pMD2G) were co-transfected into HEK293 cells. After transfection for 48 h, the viral supernatants were harvested and filtered through a 0.45 μm filter. Targeted cells were then infected with the viral supernatant and selected with puromycin (1 mg/ml) for 2 weeks. sgRNA sequences targeting PRMT5 and USP7 were designed using the CRISPR designer (http://crispr.mit.edu).

### Cell proliferation and colony-formation assay

For cell proliferation assay, indicated cells were plated in 96-well plates at 1 × 10^3^ cells per well, and then detected using CCK-8 (HY-K0301, MCE) according to the manufacture’s instruction. The absorbance was measured using a Spark® multimode microplate reader (Tecan, Männedorf, Switzerland). For colony formation assay, 500 cells were seeded into 6-well plates and cultured in medium for 10 days to 2 weeks. After macroclones (>50 cells) formed, colonies were fixed with methanol for 30 min and subsequently stained with 0.1% (m/v) crystal violet for 1 h at room temperature. The colonies were captured using microscope.

### GST pull-down assay

GST, GST-PRMT5, GST-PRMT5 truncated recombinant proteins were expressed in *Escherichia coli* BL21-DE3 by induction with 1 mM IPTG (Amersco, SF, USA). GST-tagged proteins were purified using Glutathione Sepharose 4B beads (GE Healthcare). G3BP2-His and G3BP2-His truncated proteins were purified with Ni-NTA agarose beads (Qiagen). Different purified GST- and His-tagged recombinant proteins were incubated with pull-down buffer (50 mM Tris-Cl, pH 8.0, 200 mM NaCl, 10 mM MgCl_2_, 1% NP-40, 1 mM EDTA,1 mM DTT) for 2 h at 4 °C before immunoblotting assay.

### Co-immunoprecipitation assay

Flag tagged PRMT5 full-length and its truncations were inserted into pcDNA3.1-Flag vector. The human G3BP2 cDNA were inserted into pcDNA3.1-HA vector. Indicated plasmids were transiently transfected into HEK293 cells, and after 48 h of incubation, cells were lysed in immunoprecipitation buffer as described. After washing three times, proteins in elutes were detected with anti-Flag or anti-HA antibody.

### Metabolomics analysis

Tu212 cells with ectopic G3BP2 were collected and flash-frozen in liquid nitrogen for extracting metabolites. Non-targeted liquid chromatography mass spectroscopy (LC-MS/MS) analysis and data preprocessing and annotation were performed at Shanghai Biotree Biotech Co. Ltd. Briefly, about 1000 μl extract sample (acetonitrile: methanol: water = 2: 2: 1) were homogenized and sonicated for 5 min in the ice-water bath. LC-MS/MS analyses were performed using UHPLC system (Vanquish, Thermo Fisher Scientific) with a UPLC BEH Amide column (2.1 × 100 mm, 1.7 μm) coupled to Q Exactive HFX mass spectrometer (Orbitrap MS, Thermo). The QE HFX mass spectrometer was used to acquire MS/MS spectra on information-dependent acquisition (IDA) mode in the control of acquisition software (Xcalibur,Thermo). The raw data were converted to mzXML format using ProteoWizard and processed by R package XCMS (version 3.2). R package CAMERA was used for peak annotation after XCMS data processing. The metabolites annotation were performed by accurate mass search and MS/MS spectral match using an in-house MS2 database, and adjusted *P* (FDR) values < 0.05 were considered statistically significant.

### In vitro methylation assay

5 µg of recombinant Flag-PRMT5 proteins purified from HEK293 cells or GST-G3BP2-WT or R468K proteins purified from bacteria were incubated with immunoprecipitated Flag-PRMT5 in the presence of adenosyl-L-methionine, S-[methyl-3H]. The reactions were performed in the methylation buffer (50 mM Tris pH 8.0, 20 mM KCl, 0.01% Triton X-100, 10 mM MgCl_2_, 5 mM β-mercaptoethanol, and 120 mM sucrose) at room temperature for 2 h and stopped by adding 5× SDS loading buffer and was resolved by SDS-PAGE.

### Transwell migration and invasion assay

For migration assay, 5 × 10^4^ indicated cells were plated with 300 μl serum-free medium into the uncoated or Matrigel-coated upper chamber (24-well insert, 8 μm pore size, Corning, USA) for migration or invasion assay. The bottom chamber were filled with 500 μl medium containing 20% FBS. After culturing for 36 h, the migratory or invasive cells were fixed in 4% paraformaldehyde and stained with 0.5% crystal violet solution. Five randomly selected fields from each well were imaged and counted under a microscope (magnification, ×200).

### Wound healing assay

Indicated cells were seeded in 6-well plates at a density of 2 × 10^6^ cell/well and incubated for 20 h. Then, a sterile 10-μl pipette tip was used to make the scratch line. The indicated cells were incubated with serum-free medium for 48 h. The images of each wound were observed by microscope and the wound healing rate was calculated using Image J software (http://rsb.info.nih.gov/ij/).

### Xenograft mouse model and Immunohistochemistry

4–5-week-old nude mice were purchased from Vital River (Beijing, China). The mice were randomly separated into four groups and subcutaneously injected with 1 × 10^7^ Tu686 cells, with five mice per group. Tumor volumes were measured by the formula: length × width^2^ × 0.5^2^. When the volume of xenograft tumors reached 800 mm^3^, mice were treated with indicated stable cell lines and shRNA for up to 8 weeks. Tumor volumes were measured every 3 days and at the end of the eighth weekend, and xenograft tumors were extracted and paraffin-embedded for further analysis. The animal study was conducted in accordance with protocols approved by the Animal Ethics Committee of Jiaying University.

Immunohistochemistry assay was performed as previously described [17]. Experiments using human HNSC tissues were approved by the Ethics Committee of the Sun Yat-sen University Cancer Center as described before [17]. Informed consent was obtained from the patients. The sections were incubated with primary antibodies G3BP2 (16276-1-AP; Proteintech, 1:80), PRMT5 (79998, Cell Signaling Technology, 1:50) and Ki-67 (9027, Cell Signaling Technology, 1:800). The IHC results were reviewed by two independent pathologists. The staining of G3BP2, methy-G3BP2, PRMT5 and USP7 were evaluated by IHC scores [18].

### Quantitative real-time PCR assay

RT-PCR analysis was carried out as previously described [17]. The first-strand cDNA were synthesized using a HiScript Q RT SuperMix kit (Vazyme Biotech, Nanjing, China). Quantitative real-time PCR was performed using SYBR Green Master Mix (Vazyme Biotech) on LightCycler 480 (Roche, USA). The expression levels of lipid metabolism-related genes were normalized to GAPDH. Each sample was run in triplicate. Primers used for RT-PCR in this study were listed in Supplementary Table 2.

### Statistical analysis

All statistical analysis were performed using GraphPad Prism 7.0 (GraphPad Software, USA) and SPSS 19.0 (SPSS, USA). Student’s two-tailed *t*-test was performed to analyze the significance of the differences between two groups. All data were presented as mean ± SD. Survival curves of HNSC patients were plotted by the Kaplan–Meier method. Multivariate Cox regress analyses was performed to determine independent risk factors. All data were collected from at least three independent experiments. *P* values of less than 0.05 were considered to be statistically significant.

## Results

### PRMT5 associates with and methylates G3BP2

**Fig. 1:**
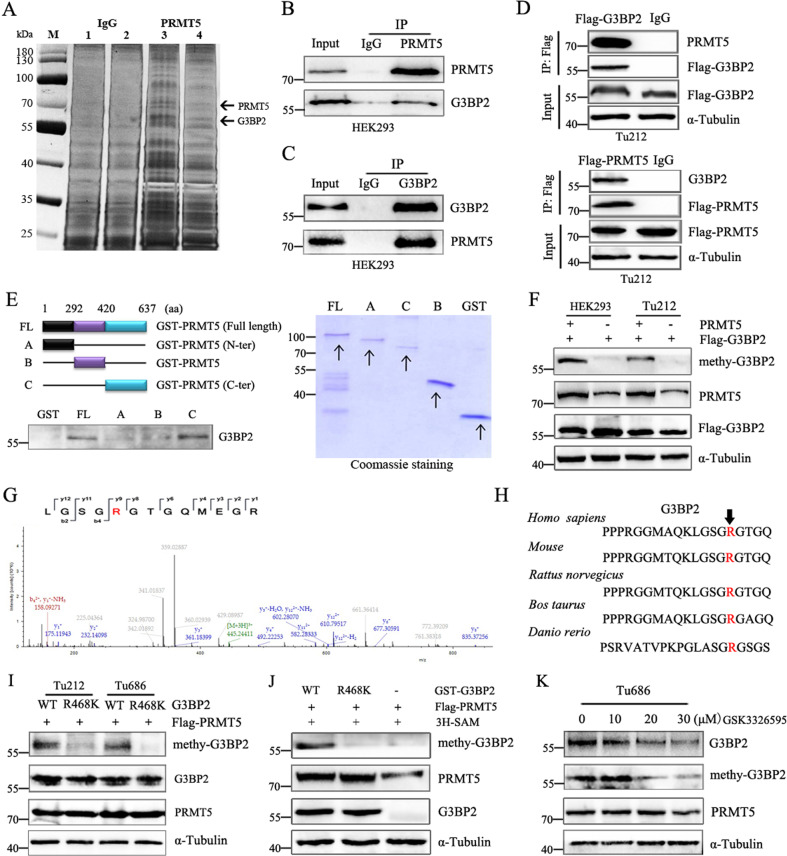
PRMT5 interacts with and methylates G3BP2. **A** PRMT5 plasmid was transfected into HEK293 and Tu686 cells. After incubated for 48 h, cell lysates were measured by Immunoprecipitation (IP) assays using anti-PRMT5 beads, and the silver staining showed the location of PRMT5 and its associated protein G3BP2. Lane 1,3 for HEK293 cell. Lane 2,4 for Tu686 cell. **B**, **C** Co-immunoprecipitation (Co-IP) of PRMT5 and G3BP2 was performed in HEK293 cells. IgG was used as a negative control. **D** Association of PRMT5 with G3BP2 were verified by Co-IP assay with anti-Flag in Tu212 cells transfected with Flag-PRMT5 or Flag-G3BP2, respectively. **E** Schematic diagram showed the structure of PRMT5 (left) and the deletion constructs were co-transfected with Flag-G3BP2 into HEK293 cells. Cell lysates were precipitated with GST beads. G3BP2 was blotted with an anti-Flag antibody. Immunoblotting and Coomassie Brilliant blue staining were shown. **F** The methylation of G3BP2 was detected by western blot analysis using a custom-made methy-G3BP2 antibody in HEK293 and Tu212 cells transfected with or without PRMT5 plasmid. **G** Methylation site of G3BP2 was examined by liquid chromatography- mass spectrometry (LC-MS). R represents potential methylation site. **H** Sequences of the evolutionarily conserved residue R468 (red) in G3BP2. **I** Validation of R468 as the PRMT5-catalyzed G3BP2 methylation site in Tu212 and Tu686 cells. **J** In vitro methylation of G3BP2 in the presence of ^3^H-SAM. Recombinant GST-G3BP2-WT and G3BP2-R468K proteins were purified from bacteria and Flag-PRMT5 proteins were immunopurified from HEK293 cells. **K** Tu686 cells were treated with the indicated amounts of GSK3326595 for 24 h, protein levels of G3BP2 and methy-G3BP2 were assessed by western blot.

Relatively few post-translational modifications of G3BP2 in human cancer cells have been reported. Our previous work identified PRMT5 is highly expressed in laryngeal cancer cells [17], and involved in regulating protein arginine methylation. To explore whether G3BP2 is a novel substrate of PRMT5, immunoprecipitation assay was performed to detect potential binding partners of PRMT5. The subsequent mass spectrometry (MS) analysis identified G3BP2, a RasGAP-binding protein, as a strong PRMT5-interacting partner (Fig. 1A). The physical association between PRMT5 and G3BP2 was validated by co-immunoprecipitation in HEK293 cells (Fig. 1B, C). In addition, we performed western blot analysis to compare the expression levels of G3BP2 and PRMT5 in HNSC cell lines (Supplementary Fig. S1A, B) and confirmed this interaction between PRMT5 and G3BP2 in Tu686 and Tu212 cells (Fig. 1D).

Next, we performed a series of truncated fragments to examine which domain of PRMT5 is responsible for the binding with G3BP2. We generated three GST-tagged truncated PRMT5 for pull-down assays, which is composed from aa 1 to 292, 293 to 420, and 421 to 637 region. The results showed that both the FL-PRMT5 and the construct with enzymatic activity region (421-637aa) could specifically interact with G3BP2 (Fig. 1E). These data strongly demonstrate that PRMT5 interacts with G3BP2, and the enzymatic activity region of PRMT5 is required for G3BP2-PRMT5 interaction.

Since GR and/or GRG repeats represent potential preferred methylation sites of PRMT5 [19], we investigate whether G3BP2 could also in fact be methylated by PRMT5. Surprisingly, PRMT5 could methylate G3BP2 as detected by a custom-made methy-G3BP2 antibody that specifically recognizes symmetric dimethyl R468 (Fig. 1F). The subsequent mass spectrometry analysis of immunopurified G3BP2 protein from HEK293 and Tu686 all suggest that arginine-468 (R468) residue was a symmetric dimethylation site (Fig. 1G and data not shown). Of note, G3BP2-R468 is evolutionarily conserved from *Rattus norvegicus* to *Homo sapiens* (Fig. 1H). Next, we verified the PRMT5 methylation site on G3BP2 by co-transfected with Flag-PRMT5 and G3BP2 into HEK293, in which the levels of G3BP2 methylation were detected. As shown in Fig. 1I, a construct containing the Arg residues at position 468 is important for PRMT5 methylates G3BP2, as R468K mutation markedly attenuated PRMT5-mediated methylation of G3BP2 compared to WT, indicating that the C-terminal GRG repeats of G3BP2 are responsible for its interaction with PRMT5. In in vitro methyltransferase assays, R468K mutants also showed lower G3BP2 methylation compared to WT (Fig. 1J). Moreover, in the presence of GSK3326595 [20], a specific inhibitor of PRMT5 methyltransferase, methylation of G3BP2 is dramatically reduced (Fig. 1K and Supplementary Fig. S1C). Taken together, these results indicated that G3BP2 interacts with and methylates by PRMT5 in an enzyme activity-dependent manner.

### PRMT5-dependent methylation of G3BP2 promotes its deubiquitination by USP7

**Fig. 2:**
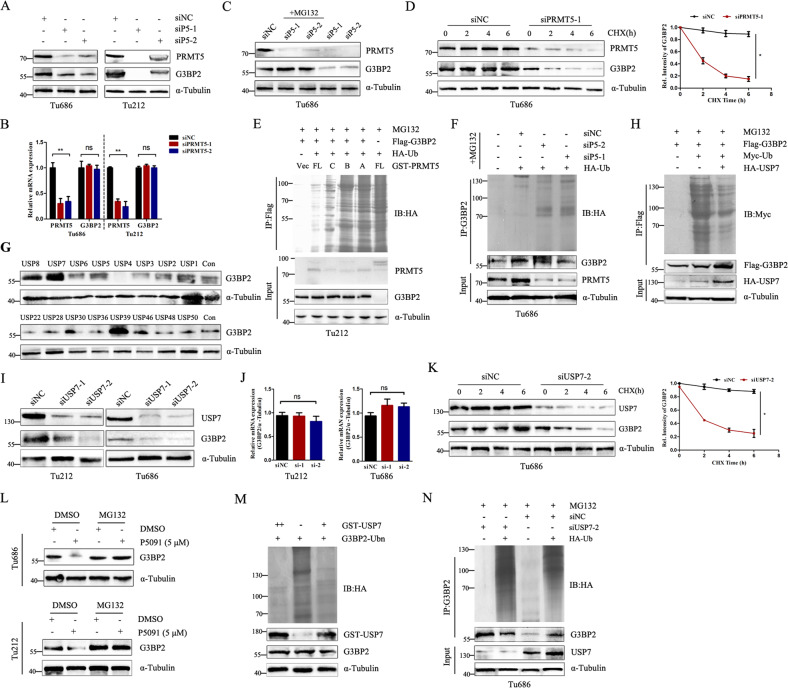
PRMT5 and UPS7 stabilizes G3BP2 by deubiquitination pathway. **A**, **B** Western blot and qPCR analysis of G3BP2 and PRMT5 expression in Tu686 and Tu212 cells transfected with siNC or siPRMT5. ns, no significant difference, **P < 0.01. **C** Tu686 cells were transfected with PRMT5 siRNAs and then incubated with or without MG132 (40 μM) for 6 h. Cell lysates were analyzed by immunoblotting. **D** Tu686 cells were transfected with negative control or PRMT5 siRNA and then applied with Cycloheximide (CHX, 50 μg/ml) for 2, 4 or 6 h. Immunoblotting analysis was used to measure the expression of G3BP2. *P < 0.05. **E** Cell lysates were immunoprecipitated with Flag-tag antibody and then immunoblotted by HA-tag antibody. **F** Cell lysates were immunoprecipitated with G3BP2 antibody before immunoblotting with HA-tag antibody. **G** HEK293 cells were transfected different deubiquitinating enzymes (DUBs) and then lysed for immunoblotting to detect the expression of G3BP2. **H** HEK293 cells were co-transfected with the indicated vectors for 48 h, followed by treated with MG132 for 6 h. Cell lysates were immunoprecipitated with anti-Flag antibody and immunoblotting with anti-Myc antibody. **I**, **J** Western blot and RT-PCR analysis of USP7 expression in Tu212 and Tu686 cells transfected with siNC or siUSP7. ns, no significant difference. **K** Tu686 cells were transfected with negative control or USP7 siRNA and then applied with 50 μg/ml CHX for the indicated times and cell lysates were assessed by immunoblotting. *P < 0.05. **L** Western blot analysis of Tu686 and Tu212 cells with or without P5091, followed by treatment with DMSO and MG132 for 6 h, respectively. **M** Tu212 cells were transfected with G3BP2 and HA-Ub plasmids for 48 h, the purified G3BP2-Ubn was added 40 ng or 80 ng recombinant GST-USP7 proteins before immunoblotting analysis. **N** USP7 knockdown in Tu686 cells increased G3BP2 ubiquitination. Tu686 cells were co-transfected with HA-Ub and USP7 siRNA or control siRNAs, followed by treated with MG132 for 6 h. Cell lysates were immunoprecipitated using an anti-G3BP2 antibody and then subjected to immunoblotting.

To investigate the mechanism of which PRMT5-mediated G3BP2 methylation affects G3BP2 expression and function, we inhibited PRMT5 expression in Tu686 and Tu212 cells. We found that knockdown PRMT5 resulted in a decrease in G3BP2 protein but not affect G3BP2 mRNA levels (Fig. 2A, B). In addition, the deubiquitinase (DUB) that protects ubiquitinated G3BP2 from degradation remains elusive. To assess the role of G3BP2 in proteasome degradation, we used the proteasome inhibitor MG-132 to determine the effect of PRMT5 depletion on G3BP2 (Fig. 2C). Likewise, we knocked down PRMT5 using specific short interfering RNAs (siRNA) and measured the half-life of G3BP2 protein. The half-life of G3BP2 protein was shorter in PRMT5-depleted cells than in the control in Tu686 cells (Fig. 2D). Furthermore, we detected the effect of PRMT5 on ubiquitination of G3BP2 by overexpressing indicated fragments into Tu212 cells. Compared with control vector and truncated group, transfection of PRMT5-WT and the construct with enzymatic region fragments significantly inhibited G3BP2 ubiquitination (Fig. 2E). However, knockdown of PRMT5 led to an enhancement of G3BP2 ubiquitination in Tu686 cells (Fig. 2F). Thus, the above results show that PRMT5 can stabilize G3BP2 by an ubiquitin-mediated pathway.

Based on the result that Arg-468 methylation decreased G3BP2 ubiquitination, we hypothesized that PRMT5 functions with an unknown DUB at post-transcriptional level to regulate G3BP2 expression. Combined with the LC-MS/MS analysis, we transfected a list of DUBs cDNA plasmids into HEK293 cells, and surprisingly found that USP7, USP8, USP39 upregulated G3BP2 expression (Fig. 2G). Of note, only USP7 of these DUBs decreased G3BP2 ubiquitination (Supplementary Fig. S2A, B). Similar observation was obtained under denaturing conditions (Fig. 2H).

Considering USP7 stabilizes its substrates, including p53, Sirt1, MDM2 [21, 22], by promoting their deubiquitination, we speculated that USP7 may affect the ubiquitination of G3BP2. To test this, we detected the effect of USP7 on G3BP2 stability. In Tu686 and Tu212 cells, USP7 depletion decreased the G3BP2 protein level, while the mRNA expression was nearly unchanged (Fig. 2I, J). The reduction of G3BP2 protein by USP7 knockdown could be due to the decreased G3BP2 stability (Fig. 2K), suggesting that USP7 stabilized G3BP2 by inhibiting its degradation through proteasome. We then evaluated whether P5091, an inhibitor of USP7, decrease G3BP2 expression. Consistently, MG132, a proteasome inhibitor, blocked P5091-induced G3BP2 decrease in both cell lines (Fig. 2L). In addition, we found that G3BP2 ubiquitination was reduced when incubating with recombinant USP7 in vitro deubiquitination analysis (Fig. 2M). Moreover, knockdown of USP7 increased G3BP2 ubiquitination in Tu686 cells (Fig. 2N). Taken together, these results suggest that USP7 regulates the stability of G3BP2 by inhibiting proteasomal degradation.

### G3BP2 deubiquitination depends on its methylation by PRMT5

**Fig. 3:**
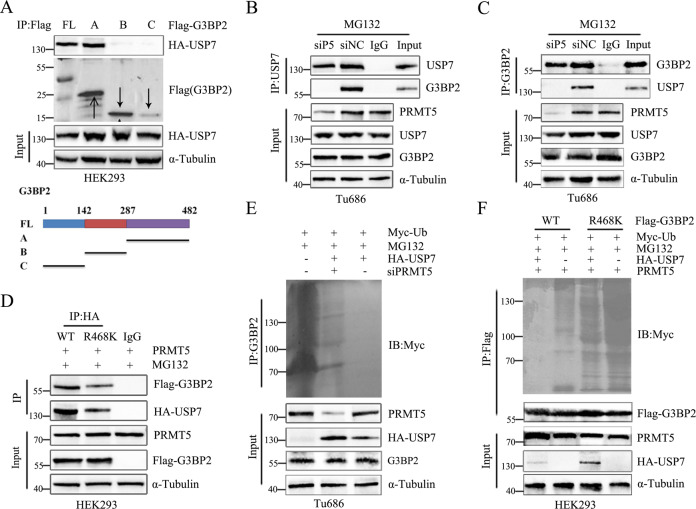
PRMT5 augment G3BP2 binding with and deubiquitination by methylation. **A** A series of G3BP2 constructs were co-transfected with HA-USP7 plasmid into HEK293 cells. Cell lysates were immunoprecipitated with anti-Flag antibody and then analyzed by immunoblotting with HA-USP7 antibody. **B**, **C** Tu686 cells were transfected with either negative control siRNA or PRMT5 siRNA, then treated with 40 μΜ MG132 for 6 h. Cell lysates were immunoprecipitated with anti-USP7 or G3BP2 antibody, followed by immunoblotting analysis. **D** Flag-G3BP2 wild type or R468K mutant was co-transfected with PRMT5 and HA-USP7 into HEK293 cells, and then treated with MG132 for 6 h. Cell lysates were immunoprecipitated with anti-HA antibody then analyzed by immunoblotting using anti-Flag antibody. **E** Cells were transfected with Myc-Ub and HA-USP7 and negative control siRNA or PRMT5 siRNA, followed by treated with MG132 for 6 h. Cell lysates were immunoprecipitated with anti-G3BP2 antibody then analyzed by immunoblotting. **F** Flag-G3BP2 wild type or R468K mutant, Myc-Ub and PRMT5 were co-transfected into HEK293 cells, and then treated with MG132 for 6 h. Cell lysates were immunoprecipitated using anti-Flag antibody and then analyzed by immunoblotting using anti-Myc antibody.

In order to confirm whether G3BP2 deubiquitination by USP7 depends on PRMT5-mediated G3BP2 methylation. We generated three truncated fragments of G3BP2, and the arginine-glycine rich motif, from 287 to 482 aa (contain PRMT5 methylation site) was required for G3BP2 interacts with USP7 (Fig. 3A), suggesting a potential link between G3BP2 methylation and deubiquitination. Accordingly, PRMT5 depletion in Tu686 cells retarded the interaction between USP7 and G3BP2 (Fig. 3B, C). G3BP2-R468K mutant significantly decreased the USP7-G3BP2 interaction in the presence of PRMT5 (Fig. 3D). In addition, the level of G3BP2 deubiquitination was attenuated after PRMT5 knockdown (Fig. 3E). Similarly, G3BP2-R468K mutation reduced the G3BP2 deubiquitination by USP7 in the presence of PRMT5 (Fig. 3F). These results demonstrate that PRMT5-dependent G3BP2 methylation is critical for G3BP2 interaction with and deubiquitination by USP7. Overall, G3BP2 acts as a central epigenetic regulatory role in the lipogenesis and PRMT5-driven laryngeal tumorigenesis.

### PRMT5-G3BP2 complex activates lipid metabolism reprogramming

**Fig. 4:**
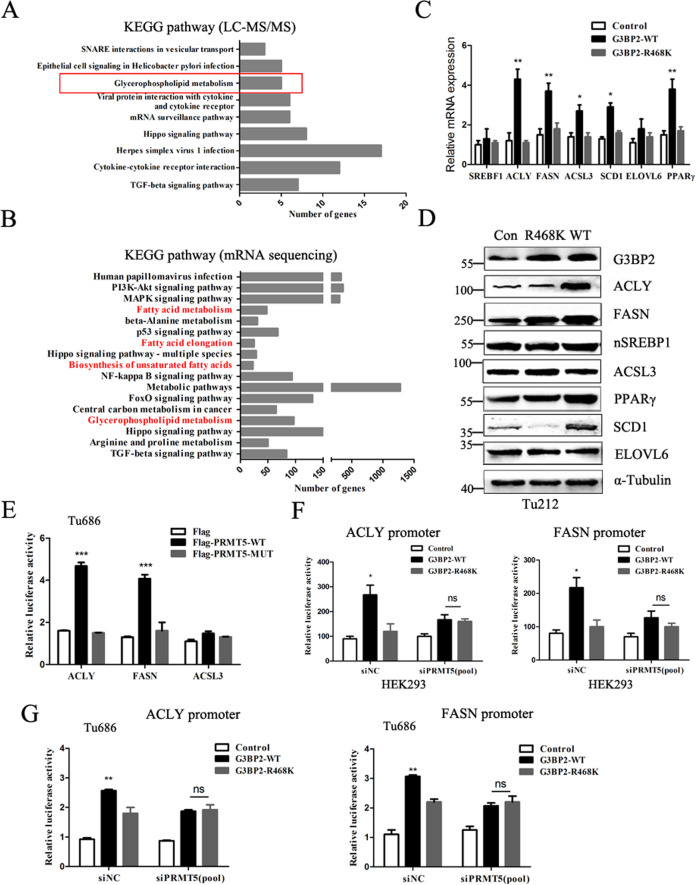
Methylation of G3BP2 by PRMT5 confers lipid metabolism reprogramming. **A** The KEGG pathway analysis of the lipid metabolism-associated pathway affected by G3BP2 in Tu212 cells. **B** The KEGG pathway enrichment analysis of G3BP2-conferred metabolism pathways in Tu212 cells. **C** The effect of G3BP2-WT or G3BP2-R468K on the expression of lipid metabolism-associated genes were measured by RT-PCR analysis in Tu212 cells. **D** The effect of G3BP2-WT and G3BP2-R468K on the lipid metabolism-associated proteins were measured by immunoblotting analysis. **E** *ACLY* and *FASN* luciferase activity were determined by co-transfected with PRMT5-WT/PRMT5-MUT and pSV-*Renilla* to Tu686 cells. Luciferase activities were measured 60 h later. ACSL3 gene was used as an negative control. **F**, **G** The G3BP2-WT or G3BP2-R468K along with ACLY and FASN luciferase reporter plasmids were co-transfected into HEK293 or Tu686-PRMT5(KO) cells for 60 h. The luciferase activities were analyzed by dual-luciferase reporter assay, and normalized to the activity of Renilla. The data are presented as the mean ± SD; ns no significant difference; *P < 0.05; ** *P* < 0.01,***P < 0.001.

To explore the biological functions of methylation G3BP2R468 on tumor cells, KEGG pathway combined untargeted metabolomics analysis were performed. As expected, Glycerophospholipid metabolism pathway belonged to lipid metabolism process was significantly enriched (Fig. 4A), suggesting that G3BP2 plays a crucial role in the lipogenesis of HNSC cells. Furthermore, RNA-seq indicated that G3BP2 conferred lipid metabolism, such as fatty acid metabolism, fatty acid elongation, biosynthesis of unsaturated fatty acids and central carbon metabolism in cancer (Fig. 4B), indicating that G3BP2 globally functions in the metabolic reprogramming of carcinomas. Then, we applied qPCR and immunoblotting analysis to confirm the effect of G3BP2 and G3BP2R468 on the expression of lipid metabolism-related genes. As shown in Fig. 4C, G3BP2 upregulated the mRNA levels of *ACLY, FASN, ACSL3, SCD1*, and *PPARγ* in Tu212 cells. Immunoblotting analysis demonstrated that G3BP2 increased the protein levels of FASN, ACLY, PPARγ, SCD1, suggesting G3BP2-WT displayed a more stronger effect than G3BP2R468 in the de novo fatty-acid biosynthesis (Fig. 4D). Next, we explored whether these lipogenic genes transcription activity were regulated by PRMT5 via the modification on R468. Consistent with previous research in lung adenocarcinoma [23], we found knockdown PRMT5 attenuated the activity of SREBP1 in Tu686 cells. Conversely, *ACLY* and *FASN* luciferase activity was dramatically increased upon ectopic expression of PRMT5 wild type but not the enzymatic inactive mutant (Fig. 4E and Supplementary Fig. S3A, B). In addition, either G3BP2-WT or R468K-mediated *ACLY* and *FASN* transcription activity was compromised by PRMT5 depletion (Fig. 4F, G). Similarly, Real-time PCR analysis showed that both of the target lipogenic genes were elevated by G3BP2-WT but not G3BP2-R468K when PRMT5 depletion (Supplementary Fig. S3C, D). These data suggested that PRMT5-G3BP2 interaction as a major co-activator complex to be recruited to the promoter of lipogenic genes for lipid metabolic reprogramming.

**Fig. 5:**
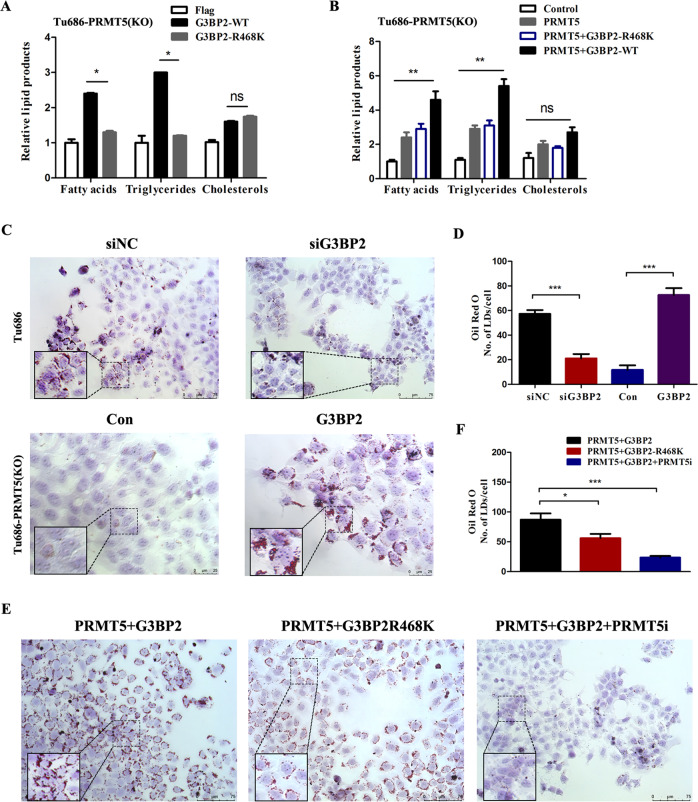
G3BP2 methylation contributes to PRMT5-induced lipids synthesis. **A**, **B** The levels of triglycerides, fatty acids and cholesterols were measured after transfected with indicated plasmids, such as pcDNA3.1-G3BP2-WT, pcDNA3.1-G3BP2-R468K, pcDNA3.1-PRMT5, pcDNA3.1-PRMT5 + G3BP2-WT or pcDNA3.1-PRMT5 + G3BP2-R468K, in PRMT5-knockout Tu686 cells. **C**, **D** The effect of G3BP2 inhibition or overexpression on lipogenesis was determined by Oil Red O staining in Tu686 and PRMT5-knockout Tu686 cells. Quantification of lipid droplets were performed by Image J. Scale bar: 75 μm, 25 μm. **E**, **F** Representative images and quantification of G3BP2 or G3BP2R468K with or without PRMT5 on lipogenesis by Oil Red O staining in PRMT5-knockout cells. Scale bar: 75 μm. PRMT5i for PRMT5 inhibitor. The data are presented as the mean ± SD; ns no significant difference, *P < 0.05; **P < 0.01,***P < 0.001.

We next tested the possible regulation of G3BP2 methylation to lipid metabolism. As expected, G3BP2-WT elevated the level of triglycerides and fatty acids, but not cholesterols in Tu686 cells, supporting that methylation of G3BP2 induces lipid metabolism reprogramming in tumor cells (Fig. 5A). PRMT5 knockout efficiency were also identified (Supplementary Fig. S4A, B). Similarly, ectopic expression of G3BP2-WT and PRMT5 elevated the triglycerides and fatty acids, but not intracellular cholesterols when compared with the G3BP2R468K group in PRMT5(KO)-Tu686 cells (Fig. 5B). To further confirm the function of G3BP2 in lipid accumulation and lipid droplets (LDs) formation, we performed Oil red O staining analysis in PRMT5 knockout Tu686 and CAL-27 cells. We found that lipid droplets formation were dramatically decreased and less abundant when G3BP2 inhibition in Tu686 and CAL-27 cells, while there were more lipid droplets formation in PRMT5(KO)-Tu686 and CAL-27 cells with G3BP2 overexpression (Fig. 5C, D and Supplementary Fig. S4C, D). Consistent with that, co-transfection of G3BP2-WT along with PRMT5 enhanced the levels of lipid droplets, suggesting that methylation of G3BP2 R468 was required for PRMT5-G3BP2 complex mediated accumulation of lipid droplets formation (Fig. 5E, F). The above results demonstrating the synergistic activation of lipogenesis by PRMT5 and G3BP2.

### Stabilization of G3BP2 by PRMT5 and USP7 promotes tumorigenesis of carcinoma cells

**Fig. 6:**
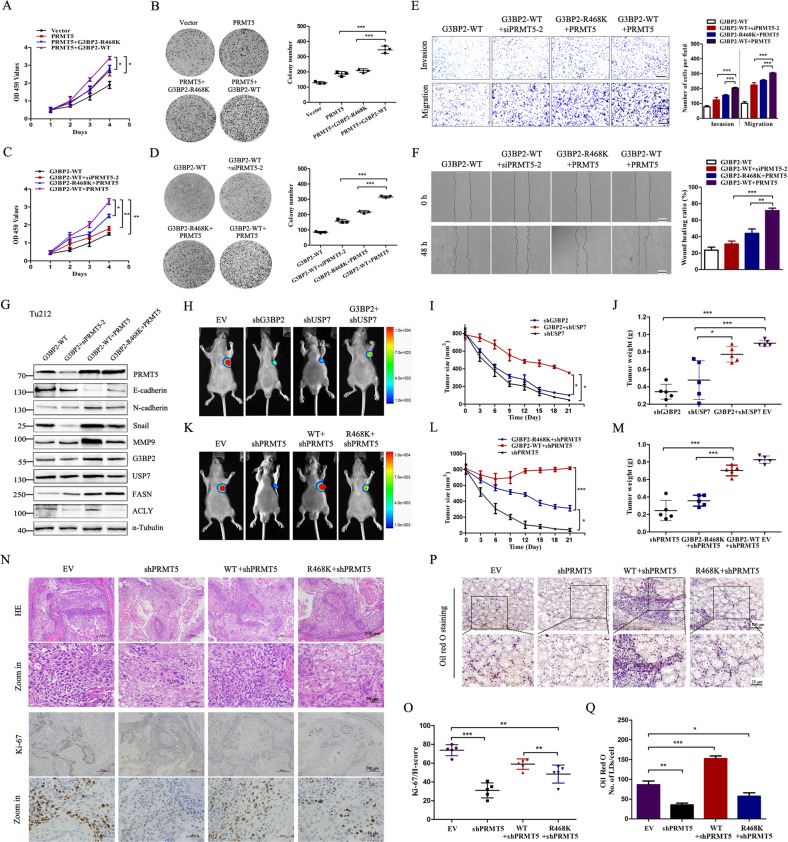
PRMT5-USP7-G3BP2 axis is required for tumorigenesis of HNSC. **A**, **C** Cell viability was analyzed by Cell counting kit-8 (CCK-8). **B**, **D** Colony formation assays were performed to evaluate the proliferation ability of the cells with indicated treatments. Cells were seeded into six-well plates at a density of 1000 cells/well, and cultured for 12 d, and then stained with crystal violet. The colonies were captured and counted. **E** Transwell migration and invasion assays were used to estimate the migration and invasion ability of G3BP2-WT and G3BP2-R468K with or without PRMT5 expression. Representative pictures are shown on the left, and quantifications on the right. Scale bar: 100 μm. **F** Representative pictures shown the wound healing assays of G3BP2-WT and G3BP2-R468K with or without PRMT5 expression. Scale bar: 100 μm. **G** Immunoblotting analysis validated the protein expression levels of EMT and lipid metabolic-related markers. **H** Representative images of tumor-bearing nude mice. Tu686 cells were treated as indicated, such as EV, shG3BP2, shUSP7, and G3BP1 + shUSP7, and then injected into nude mice (*n* = 5). **I** Tumor diameters were measured every 3 days, and tumor volumes were calculated. **J** Average tumor weight of the xenografts in each group were weighed. **K** Representative images of tumor-bearing nude mice. G3BP2-WT and G3BP2-R468K overexpression Tu686 cells with shPRMT5 were respectively injected into nude mice (*n* = 5). **L**, **M** Tumor diameters and tumor volumes were calculated. **N** The levels of proliferation marker of Ki-67 were examined in different groups of tissues. Scar bar: 200 μm; 50 μm. **O** The H score of Ki-67 in different groups. **P** Representative images of Oil red O staining of tumor tissues from nude mice. scale bar: 100 μm; 25 μm. **Q** The quantification of the number of lipid droplets per cell among 200 cells in different groups. The data are presented as the mean ± SD *P < 0.05, **P < 0.01, ***P < 0.001.

Previous studies have shown that higher levels of G3BP2 status correlated with malignant progression and poor prognosis [7], we sought to confirm if it also occurs in a methylation-dependent way. Hence, the function of PRMT5-G3BP2 interaction on tumorigenesis was further explored. In the USP7-stable Tu686 cell lines, CCK-8 and colony formation assays showed that the ectopic expression of G3BP2-WT increased PRMT5-mediated cell proliferation compared with the G3BP2-MUT (Fig. 6A, B). Additionally, the enhanced proliferation induced by overexpression of G3BP2-WT was blocked when the absence of PRMT5, suggesting that the methylation of G3BP2 is involved in the event of PRMT5 promotes carcinoma cell growth (Fig. 6C, D). Moreover, transwell and wound healing assays were used to confirm this observation. G3BP2-WT and PRMT5 co-transfected cells dramatically promoted the migration and invasion, whereas the G3BP2-R468K + PRMT5 and G3BP2-WT + siPRMT5 group cells presented a little bit faster migration capacity than G3BP2-WT cells (Fig. 6E, F). Meanwhile, we measured a series of metastasis-related genes, which were reported to be associated with epithelial-mesenchymal transition (EMT). The results revealed that G3BP2-R468K and PRMT5 apparently abolished the expression levels of EMT-related genes (Fig. 6G). Thus, we conclude that methylation of G3BP2 R468 promotes the proliferation and migration of cancer cells in vitro.

A xenograft model was established to further explore whether PRMT5 and USP7 regulate the tumorigenesis through G3BP2. To this end, we performed G3BP2-WT and the methylation-deficient mutant G3BP2-R468K stable cell lines in Tu686 cells. Notably, depletion of G3BP2 and USP7 independently retarded tumor growth, but the efficacy of USP7 silencing was rescued by G3BP2 expression (Fig. 6H–J). Next, we pinpoint the role of PRMT5-dependent methylation of G3BP2-R468 in vivo. Compared to G3BP2-R468K mutation, G3BP2-WT dramatically rescued the inhibitory effect of PRMT5 silencing on tumor growth (Fig. 6K–M). IHC analysis was performed to observe the proliferation of tumors by using an antibody against Ki-67. As expected, tumors derived from G3BP2-R468K group were less proliferative than those from G3BP2-WT (Fig. 6N, O and Supplementary Fig. S5). Oil red O staining assay was carried out to evaluate the lipid accumulation in tumors, whereas G3BP2-WT rescued PRMT5 silencing-mediated intracellular lipid accumulation (Fig. 6P, Q). Altogether, PRMT5-mediated G3BP2-R468 methylation is an important step for its activation and oncogenic function in HNSC cell models.

### G3BP2 expression is correlated with PRMT5 and USP7 in HNSC specimens

**Fig. 7:**
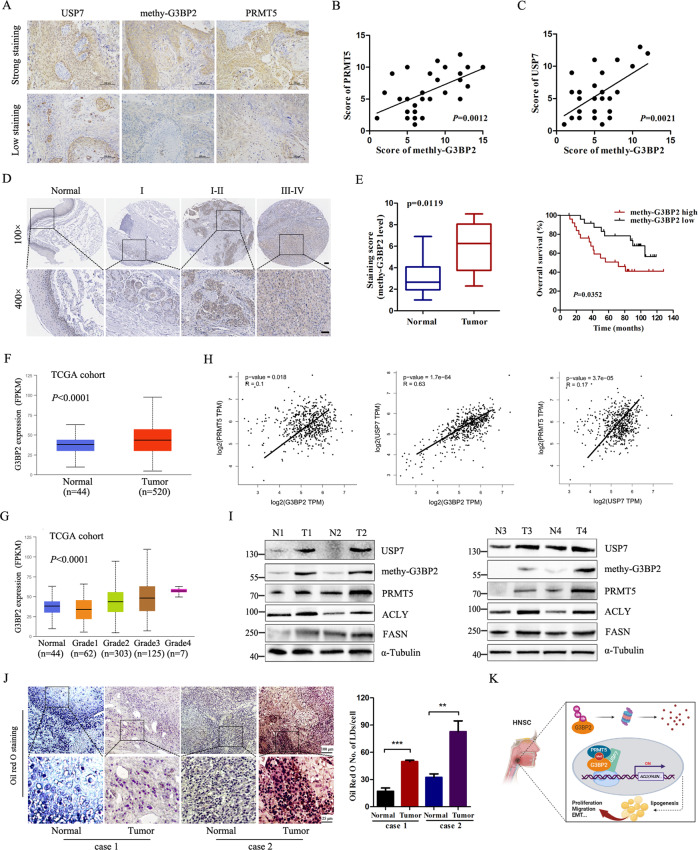
Methylated G3BP2 is up-regulated in HNSC tissues and predicts poor prognosis. **A** Representative IHC images of methy-G3BP2, USP7, and PRMT5 proteins in HNSC tumor samples. Scale bar: 100 μm. Histogram shows the H score of methy-G3BP2 with PRMT5 (**B**) and USP7 (**C**), note that the scores of some samples overlapped. **D** IHC analysis estimate the expression of methy-G3BP2 in different tumor grade of HNSC. **E** Kaplan–Meier analyses of HNSC specimens survival stratified by the expression of methy-G3BP2. **F** mRNA expression of G3BP2 in HNSC tissues and normal tissues from the TCGA database. **G** Correlation between *G3BP2* expression and tumor grade of HNSC patients in the TCGA cohort. **H** Pearson correlation coefficient between G3BP2, USP7, and PRMT5 in TCGA-HNSC cohort. **I** Immunoblotting analysis of indicated proteins expression level in HNSC and corresponding adjacent non-tumor tissues. **J** Representative images and quantification of the number of lipid droplets per cell among 200 cells of Oil red O staining in tumor and non-tumor tissues. **P < 0.01, ***P < 0.001. **K** Proposed model to describe the role of G3BP2-R468 methylation on promoting USP7- and PRMT5-dependent lipogenesis and tumorigenesis.

The results of G3BP2 methylation by PRMT5 promotes HNSC cells de novo lipogenesis and growth capacity prompted us to explore whether the methylation of G3BP2R468, PRMT5, and USP7 are positively correlated in HNSC tissues. 50 pairs of HNSC samples with adjacent normal tissues were analyzed by IHC staining using an antibody against R468-G3BP2. As shown in Fig. 7A, the majority of HNSC biopsies displayed PRMT5, USP7 and G3BP2-positive staining in both cytoplasmic and nuclei of HNSC tissue cells, whereas weak diffused in adjacent normal HNSC tissue cells. H score analysis showed a significant higher expression levels of PRMT5, USP7 and methylated G3BP2 in HNSC tissues compared with adjacent normal tissues (Fig. 7B, C). Strikingly, we observed the levels of methylated G3BP2 was correlated with tumor grade of HNSC specimens, and survival analysis suggested that elevated levels of methy-G3BP2 associated with poor overall survival (Fig. 7D, E). Similarly, we validated that the expression of G3BP2 was higher in HNSC than normal tissues in the TCGA-HNSC cohort, and high G3BP2 expression associated with tumor grade (Fig. 7F, G). Likewise, the USP7 and PRMT5 expression was also increased in the TCGA-HNSC tissues than normal tissues (Supplementary Fig. S6A, B). Moreover, the relationship between methy-G3BP2 expression and clinicopathological factors was summarized in Supplementary Table 3. In addition, Pearson’s correlation analysis was performed to validate the association of PRMT5, USP7, and G3BP2 in TCGA-HNSC samples (Fig. 7H). Immunoblotting assays verified the upregulation of methy-G3BP2, PRMT5, USP7 and lipid metabolism-related proteins in HNSC samples. The methy-G3BP2 levels was positive correlated with the levels of ACLY, but not FASN (Fig. 7I). Oil red O staining assay was performed to estimate the intracellular lipid droplet formation, the accumulation lipid droplets were significantly much more abundant in the tumors (Fig. 7J). All these findings suggested that PRMT5-dependent methylation of G3BP2 is partly responsible for the tumorigenicity of HNSC. Dysregulated G3BP2 expression in HNSC is driven by high-level PRMT5 and USP7. Based on these results, we propose an epigenetic de novo lipogenesis and tumorigenesis model (Fig. 7K). The ectopic expression of PRMT5 interacts with G3BP2, G3BP2 in turn deubiquitination and stabilization by USP7, and then recruitment of methy-G3BP2-PRMT5-USP7 complex to lipogenic gene promoters, thereby activating lipogenesis and tumorigenesis.

## Discussion

Hyperactivation of the metabolic reprogramming, including *de no* lipogenesis pathway is a hallmark of human cancers, which can be caused by genetic alterations or post-translational modifications [24, 25]. Notably, increased lipid synthesis in HNSC has been observed; however, the underlying molecular mechanisms remain not fully investigated. In this study, we performed experimental evidence showing that USP7-mediated G3BP2 deubiquitination and stabilization is attenuated by PRMT5 depletion or chemical inhibition of methyl-transferase activity of PRMT5. PRMT5 and USP7 serve as a G3BP2-sensitive ‘switch’ in regulating its stabilization and lipid metabolism reprogramming, which leads to lipogenesis and aggressive malignancy of HNSC. The PRMT5-USP7-G3BP2 signal is critical for tumorigenesis, which provides a potential rationale for further pharmacological study as well.

G3BP2 was preliminary identified as an androgen-responsive gene and consists of a nuclear transport factor 2-like domain, an RNA recognition motif, and an arginine and glycine rich region [26]. Of note, G3BP2 is relatively poorly documented when compared with G3BP1, another G3BPs family members. In the current study, we confirmed that G3BP2 binds to PRMT5 via its arginine and glycine rich box motif. The C-terminal GRG repeats of G3BP2 are required for their interactions. As RNA binding proteins have been engaged in posttranscriptional modification by modulating RNA splicing, mRNA localization and stability [27, 28]. Therefore, RNA binding proteins are essential for maintaining homeostasis of gene expression. As a RNA binding protein, abnormal expression of G3BP2 exerts critical roles in gene expression homeostasis and cause tumor progression [4, 8, 29]. In the present study, we demonstrate that G3BP2 was methylated at R468 to allow direct interaction with USP7 and via an enhance in *ACLY* and *FASN* transcription levels, thereby methylation-stabilized G3BP2 increased de novo lipogenesis and accelerated the growth of HNSC cells in vivo and in vitro. Moreover, we identified G3BP2 as an independent prognostic marker in HNSC, consistent with prior reports in prostate cancer, breast and lung cancer [8, 9, 30]. Importantly, we suggest G3BP2 is involved in PRMT5-dependent activation of the lipogenic promoter in HNSC cells, and the repression of G3BP2 decreases triglyceride levels. We ascertained G3BP2-dependent growth induction was eliminated by USP7 inhibition. G3BP2 methylation by PRMT5 promotes G3BP2 binding with and then deubiquitination by USP7, which provides a novel epigenetic link between substrates methylation and deubiquitination.

As a well-known oncogenic enzyme, PRMT5 is ubiquitously expressed in the cytoplasm and nucleus of carcinoma cells. The function of PRMT5 in malignancy is due to the context with substrate and tumor types. PRMT5 dysregulation has been involved in cellular hyperproliferation, metastasis, apoptosis, EMT and differentiation [31]. We previously reported that PRMT5 is highly expressed in laryngeal carcinoma, of which the most malignant subtype of HNSC [17]. PRMT5 catalyzes a series of substrates, including histone and non-histone proteins [32–34]. Its methylation modification of arginine residues has been linked to several cellular processes, such as cell growth, differentiation, and lipid metabolism [35, 36]. PRMT5 arginine methylation of p53 gene was required in altered nuclear localization and activation in promoting lymphomagenesis [37, 38], therefore, we speculated if PRMT5 was involved in the lipogenesis through epigenetic modification. Through mRNA-seq and KEGG analysis, we suggested that overexpression PRMT5 activates head and neck squamous carcinoma lipid metabolism pathway. Here, we found ectopic expression of PRMT5 could interact and methylate G3BP2 dramatically increased HNSC progression. The data from the TCGA database in addition to our cohort strongly show that PRMT5 and G3BP2 are frequently up-regulated in HNSC tissues. Notably, our data show that depletion the enzyme activity of PRMT5 by GSK3326595, which is being tested in clinical trials, dramatically abrogate the methylation of G3BP2. These findings indicate that G3BP2 methylated by PRMT5 is in an enzyme activity-dependent manner. Consistent with our results, both PRMT5 and G3BP2 have emerged as crucial therapeutic targets for several diseases [39–41]. However, it is expected that whether different types of PTMs on the same protein will exhibit different effects, which ensure cancer cells effectively coordinate metabolic regulation in order to maximize their survivability.

Ubiquitination, a reversible process, is driven by a series of enzymes in most eukaryotic cells. Protein deubiquitination is well certified to be reserved by deubiquitinating enzymes. Regulation of G3BP2 ubiquitination and deubiquitination in cancer cells is of great interest but remains unclear. Our present study identified that G3BP2 methylation affects its recognition by deubiquitinating enzymes (DUBs). USP7, a member of ubiquitin-specific processing proteases, was regarded as a biomarker for predicting metastasis and recurrence of several malignant tumors [42–44]. USP7 regulates p53 and its E3 ligase MDM2 by preventing their degradation in order to control protein network [45]. USP7 could also deubiquitinate and stabilize EZH2 in prostate cancer cells [46]. As far as we know, little is known about the function of USP7 in HNSC tumorigenesis and elucidated new substrates of USP7 is of great interest. Our present study identified that methylation G3BP2 by PRMT5 increased its binding with and deubiquitination by USP7. Moreover, USP7 was highly expressed in HNSC, and was recruited to the *ACLY* gene promoter, in which USP7 stabilizes G3BP2 and epigenetically enhancing lipogenic genes expression and lipogenesis. In contrast, other deubiquitinases such as USP10, which is reported to mediate G3BP2/G3BP1 deubiquitination has no correlation with G3BP2R468me2 under our system [7]. Recent studies reported that USP7 acts as a co-activator in tumor initiation by stabilizing Axin and hnRNPA1 [47, 48]. Consistent with these results, our study indicates that abolish USP7 expression retards tumor growth of HNSC cells. Our present study identified that G3BP2 methylation by PRMT5 promotes G3BP2 binding with and then deubiquitination by USP7, which suggests a link between substrates methylation and deubiquitination. Despite considerable analysis arranged in this study on the USP7 and G3BP2 regulatory circuit, it is not yet fully clear how USP7-G3BP2 promoted lipid metabolic reprogram cells to help the cancer state.

Our results extend the knowledge regarding of G3BP2 methylation and stabilization for tumor progression. High-level PRMT5 and USP7 are responsible for the accumulation of, and enhancing G3BP2 deubiquitination, which leads to lipogenesis and aggressive malignancy of HNSC. A PRMT5-USP7-G3BP2 regulatory complex is a potential therapeutic strategy for HNSC and other lipid metabolic diseases as well.

## Supplementary information

Supplementary tables

Supplementary figure legends

Supplementary Figure S1

Supplementary Figure S2

Supplementary Figure S3

Supplementary Figure S4

Supplementary Figure S5

Supplementary Figure S6

checklist

Original Data File

## Data Availability

The data that support the findings of this study are available from the corresponding author upon reasonable request.
