# Supplementary material for: USP7- and PRMT5-dependent G3BP2 stabilization drives de novo lipogenesis and tumorigenesis of HNSC
Source: Cell Death Dis. 2023 Mar 6;14(3):182. doi: 10.1038/s41419-023-05706-2 (PMC9988876; doi:10.1038/s41419-023-05706-2)
Supplement: Supplementary file 1 — Supplementary tables [file 41419_2023_5706_MOESM1_ESM.docx]

**Table S1. siRNA and shRNA sequences**

| Gene | Sequences |
| --- | --- |
| G3BP2 shRNA | CGGGAGTTTGTGAGGCAATAT  GACTCTGACAACCGTAGAATA |
| G3BP2 siRNA | AGUCGAAGCUAAACCAGAA  UUCUGGUUUAGCUUCGACU |
| PRMT5 siRNA | GCACCAGUCUGUUCUGCUA  GGACCUGAGAUGAUAUA |
| PRMT5 shRNA | CCCATCCTCTTCCCTATTAAG  GCCCACTTTGAGATGCCTTAT |
| USP7 shRNA | TCTTCAGCACTGCTTGTGCA  GAGTGAGTGGACAACACCG |
| USP7 siRNA | UAAGGACCCUGCAAAUUAU  GCAUAGUGAUAAACCUGUA |

**Table S2. Quantitative real-time PCR primers**

| Gene | Forward primer | Reverse primer |
| --- | --- | --- |
| FASN | GTCGGGCCAATCCGACCACG | TATCAACAAGCATTCCACCG |
| ACLY | TGGACCTACAGTCACCTCGG | ACCATACGCAGTAGTCATCG |
| SREBF1 | ATACGCAGTAGTCATCGTGG | GAGTCCTTCCACGATACC |
| ACLS3 | GCACCCAGCTCGAGAGGCATA | GTGACTAACTATGCAATAATCCACC |
| SCD1 | TGTGCACGAAGGACAGCCCCT | CCTGCTCTGCAGTGAGAGGGA |
| EVOVL6 | GGGTCCTTTTCAAACACTTCA | GAGCGACGAGATGGACAATAAGA |
| PPARγ | GAAGTCCATGGGGCACCACCA | CTGAGACCTGGGTACACGCTG |
| GAPDH | GTCTCCTCTGACTTCAACAGCG | ACCACCCTGTTGCTGTAGCCAA |
| G3BP2 | CCTCGTGTGCGTGAACAAC | CATGTGGCAAGTTACCAACAAAA |

| Table S3. The associations between methy-G3BP2 expression and clinicopathological parameters in patients with HNSC | | | | | |
| --- | --- | --- | --- | --- | --- |
| Characteristics |  | **n methly-G3BP2 expression**  **high(n=48) low(n=40)** | ***P-*value** |  |  |
| \| **Age(years)** \|  \|  \|  \| 0.4982 \| \| --- \| --- \| --- \| --- \| --- \| \| < 50 \| 22 \| 9(40.9%) 13(59.1%) \|  \|  \| \| > 50 \| 28 \| 13(46.4%) 15(53.6%) \|  \|  \| \| **Gender** \|  \|  \|  \| 0.4117 \| \| Male \| 40 \| 28(70.0%) 12(30.0%) \|  \|  \| \| Female \| 10 \| 5(50.0%) 5(50.0%) \|  \|  \| \| **Tumor size** \|  \|  \|  \| 0.3927 \| \| <5 cm \| 25 \| 13(52.0%) 12(48.0%) \|  \|  \| \| >5 cm \| 25 \| 15(60.0%) 10(40.0%) \|  \|  \| \| **Smoking** \|  \|  \|  \| 0.8615 \| \| No \| 30 \| 17(56.7%) 13(43.3%) \|  \|  \| \| Yes \| 20 \| 11(55.0%) 9(45.0%) \|  \|  \| \| **Alcohol use** \|  \|  \|  \| 0.2401 \| \| No \| 24 \| 16(66.7%) 8(33.3%) \|  \|  \| \| Yes \| 26 \| 12(46.2%) 14(53.8%) \|  \|  \| \| **Differentiation** \|  \|  \|  \| 0.1737 \| \| Well \| 23 \| 11(47.8%) 12(52.2%) \|  \|  \| \| Moderate-Poor \| 27 \| 17(63.0%) 10(37.0%) \|  \|  \| \| **T-primary tumor** \|  \|  \|  \| **0.0306** \| \| T_1+2_ \| 16 \| 4(25.0%) 12(75.0%) \|  \|  \| \| T_3+4_ \| 34 \| 18(52.9%) 16(47.1%) \|  \|  \| \| **Metastases** \|  \|  \|  \| 0.3907 \| \| No \| 17 \| 8(47.1%) 9(52.9%) \|  \|  \| \| Yes \| 33 \| 10(30.3%) 23(69.7%) \|  \|  \| \| **TNM stage** \|  \|  \|  \| **0.0168** \| \| I-II \| 27 \| 12(44.4%) 15(55.6%) \|  \|  \| \| III-IV \| 23 \| 17(73.9%) 6(26.1%) \|  \|  \|     Note: Bold value showed significance. | | | |  | 0 |
