# Supplementary material for: USP7- and PRMT5-dependent G3BP2 stabilization drives de novo lipogenesis and tumorigenesis of HNSC
Source: Cell Death Dis. 2023 Mar 6;14(3):182. doi: 10.1038/s41419-023-05706-2 (PMC9988876; doi:10.1038/s41419-023-05706-2)
Supplement: Supplementary file 2 — Supplementary figure legends [file 41419_2023_5706_MOESM2_ESM.docx]

**Supplementary Figure 1 A** Western blot analysis of G3BP2 and PRMT5 expression in different HNSC cell lines compared to a Normal Oral Keratinocytes cell line (NOK). **B** Association of PRMT5 with G3BP2 were varified by Co-IP assay with anti-Flag in Tu686 cells transfected with Flag-PRMT5 or Flag-G3BP2, respectively. **C** Tu212 cells were treated with the indicated amounts of GSK3326595 for 24 h, protein levels of G3BP2 and methy-G3BP2 were assessed by Western blotting.

**Supplementary Figure 2** USP7 deubiquitinates and stabilizes G3BP2. **A** Effect of DUBs on the ubiquitination of G3BP2. HEK293 cells were transfected with Flag-G3BP2, Myc-Ub, and USP7, USP8, or USP39 and then treated with MG132 for 6 h before harvest. Cell lysates were immunoprecipitated using an anti-G3BP2 antibody and immunoblotting analysis using the indicated antibodies. **B** HEK293 cells were transfected with Flag-G3BP2, Myc-Ub, and HA-USP39 or Flag-USP7. Cell lysates were immunoprecipitated with Flag-G3BP2 antibody (left) or Myc antibody (right).

**Supplementary Figure 3** Methylation of G3BP2 on R468 by PRMT5 promotes lipid metabolism-related genes transcriptional activity. **A,B** HEK293 cells were co-transfected with Flag-PRMT5-WT or mutant Flag-PRMT5-A, B or C (or empty vector as the control) for 48 h before luciferase reporter assay. The mRNA levels of *ACLY* (**C**) and *FASN* (**D**) in HEK293 cells transfected with shRNA-PRMT5 or shRNA-negative control (NC) for 48 h were performed by real-time PCR. The data are presented as the mean ± SD; **p*< 0.05; ***p*< 0.01.

**Supplementary Figure 4 A** T7E1 digestion of Cas9 cutting efficiencies. **B** Protein extracts of Tu686 cells expressing PRMT5 and USP7 were analyzed by immunoblotting. **C,** **D** The effect of G3BP2 inhibition or overexpression on lipogenesis was determined by Oil Red O staining in CAL-27 and CAL-27 –PRMT5(KO) cells. The data are presented as the mean ± SD; ***p*< 0.01.

**Supplementary Figure 5** Representative images of IHC staining for PRMT5, G3BP2 and USP7 in tumor tissues from nude mice.

**Supplementary Figure 6** USP7 and PRMT5 are up-regulated in TCGA-HNSC cohort. **A** mRNA expression of USP7 in HNSC tissues and normal tissues from the TCGA database (left panel), and correlation between *USP7* expression and tumor grade of HNSC patients in the TCGA cohort (right panel). **B** mRNA expression of PRMT5 in HNSC tissues and normal tissues from the TCGA database (left panel), and correlation between *PRMT5* expression and tumor grade of HNSC patients in the TCGA cohort (right panel).
