# Supplementary figures and images for: USP7- and PRMT5-dependent G3BP2 stabilization drives de novo lipogenesis and tumorigenesis of HNSC
Source: Cell Death Dis. 2023 Mar 6;14(3):182. doi: 10.1038/s41419-023-05706-2 (PMC9988876; doi:10.1038/s41419-023-05706-2)

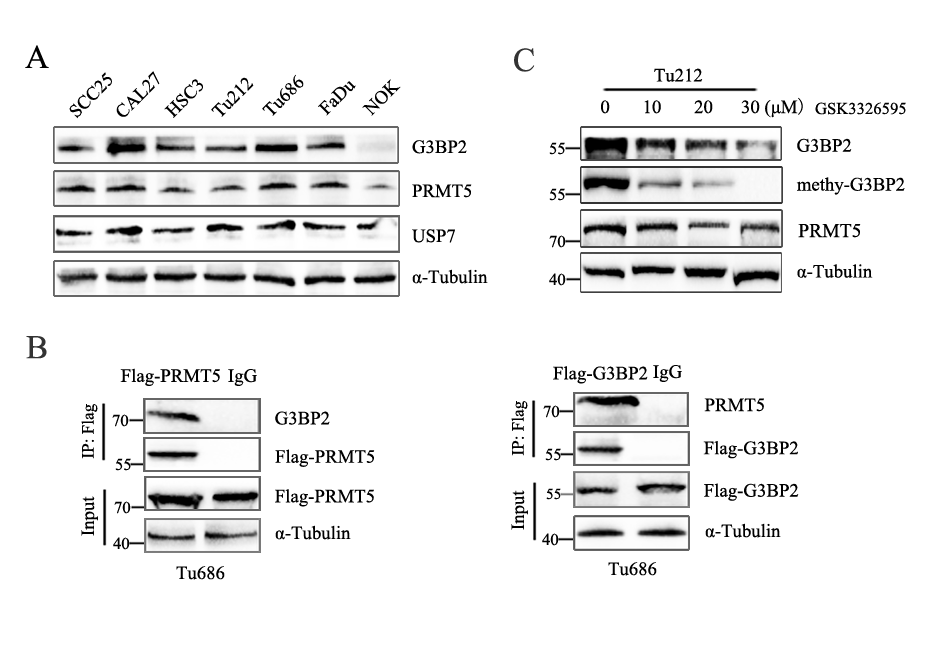

Supplement: Supplementary file 3 — Supplementary Figure S1 [file 41419_2023_5706_MOESM3_ESM.tif]

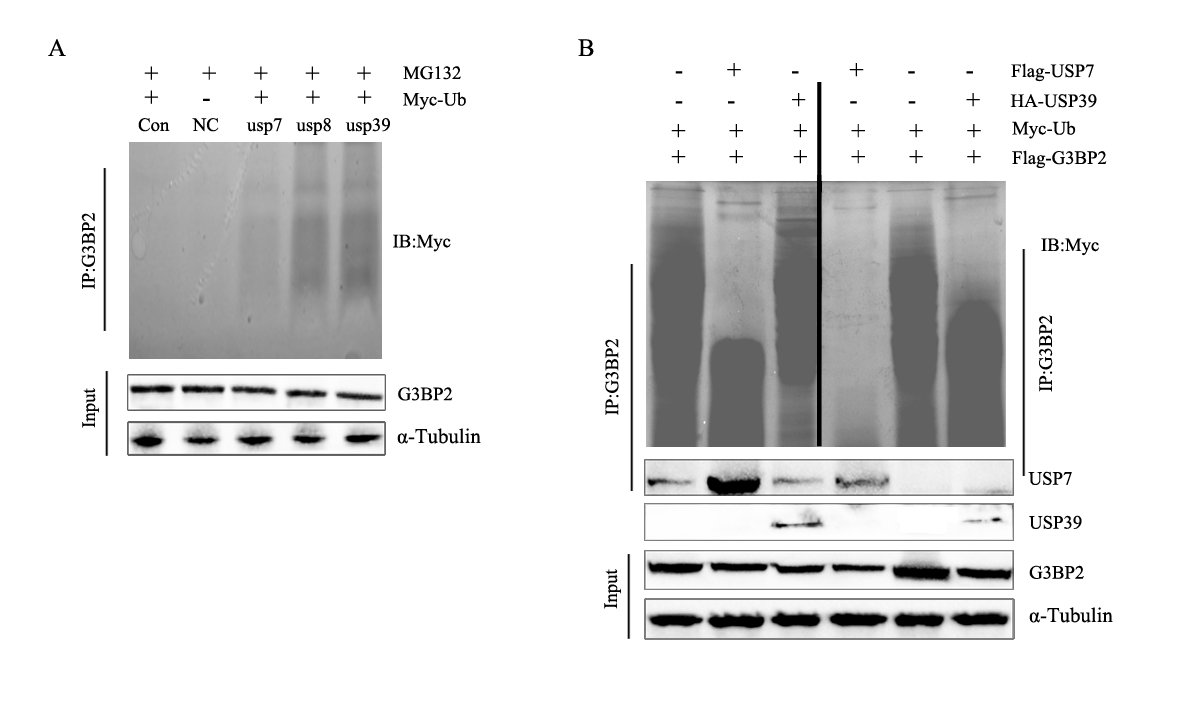

Supplement: Supplementary file 4 — Supplementary Figure S2 [file 41419_2023_5706_MOESM4_ESM.tif]

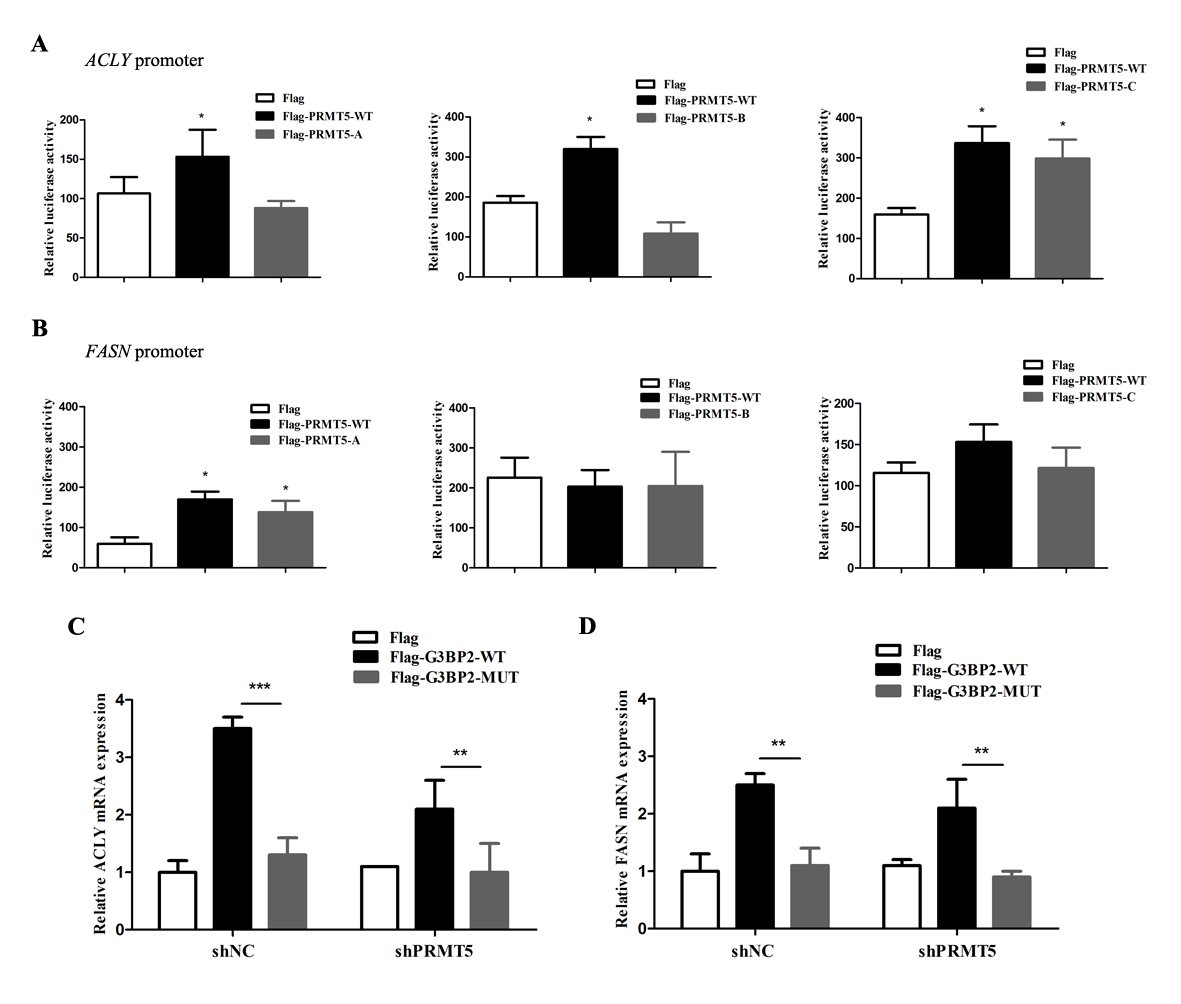

Supplement: Supplementary file 5 — Supplementary Figure S3 [file 41419_2023_5706_MOESM5_ESM.tif]

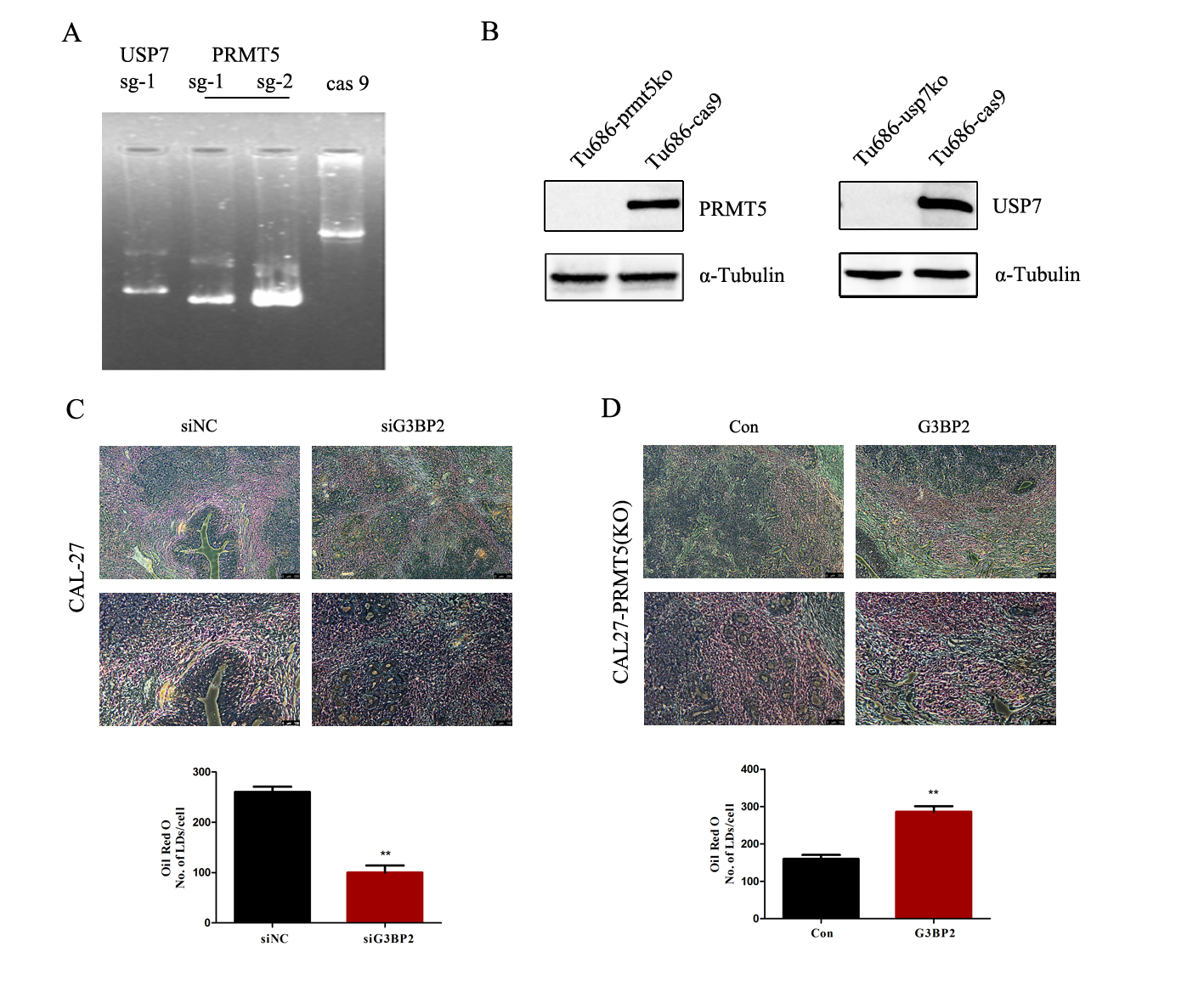

Supplement: Supplementary file 6 — Supplementary Figure S4 [file 41419_2023_5706_MOESM6_ESM.tif]

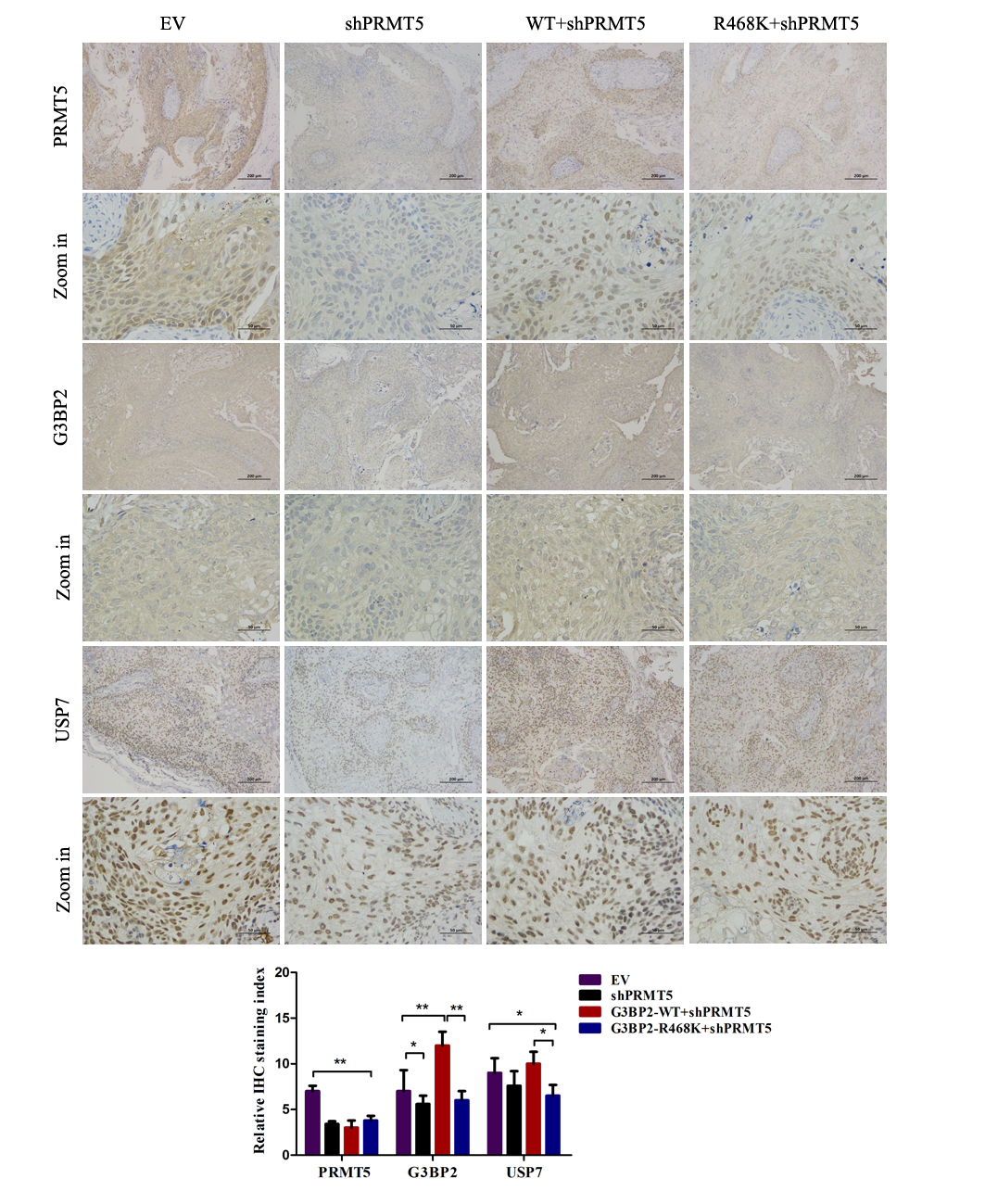

Supplement: Supplementary file 7 — Supplementary Figure S5 [file 41419_2023_5706_MOESM7_ESM.tif]

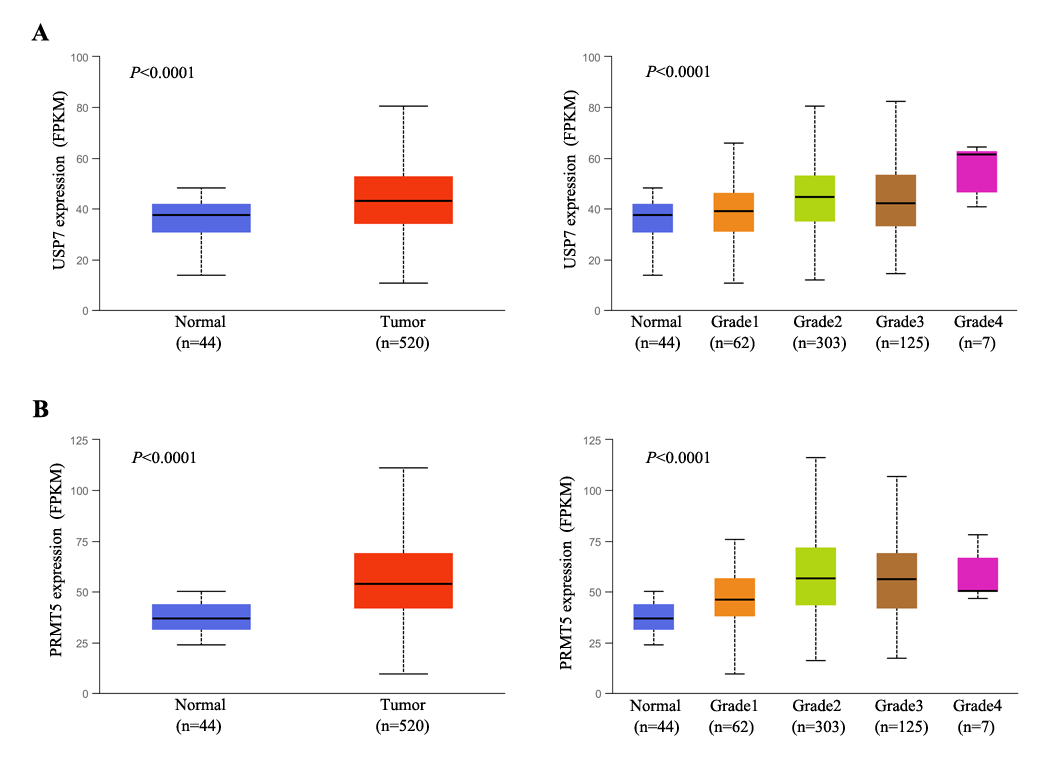

Supplement: Supplementary file 8 — Supplementary Figure S6 [file 41419_2023_5706_MOESM8_ESM.tif]
