# Supplementary material for: USP7- and PRMT5-dependent G3BP2 stabilization drives de novo lipogenesis and tumorigenesis of HNSC
Source: Cell Death Dis. 2023 Mar 6;14(3):182. doi: 10.1038/s41419-023-05706-2 (PMC9988876; doi:10.1038/s41419-023-05706-2)
Supplement: Supplementary file 10 — Original Data File [file 41419_2023_5706_MOESM10_ESM.pdf]

Fig.1		
	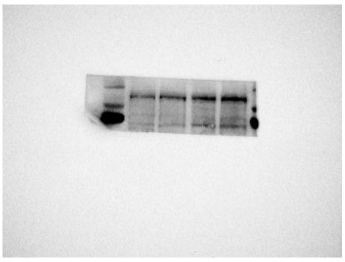	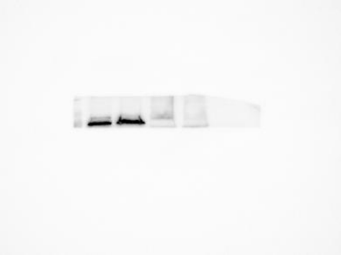
Flag-G3BP2	G3BP2 (2)	G3BP2 (3)
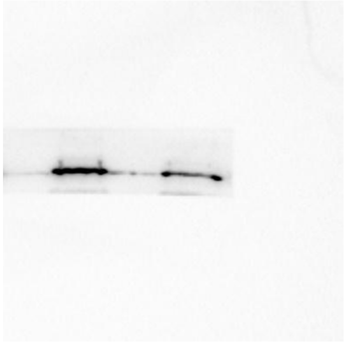		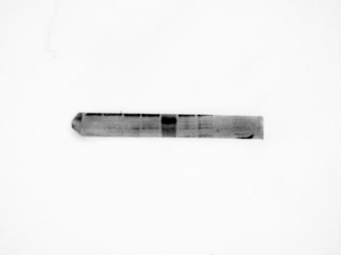
G3BP2	G3BP2	GAPDH
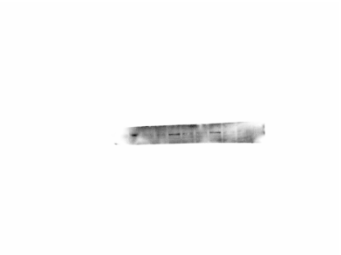	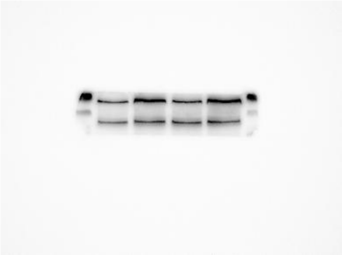	
	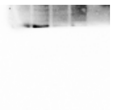	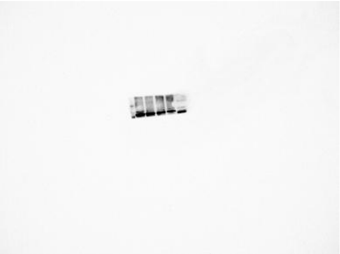
	methy G3BP2	p5

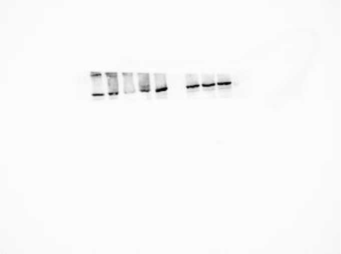		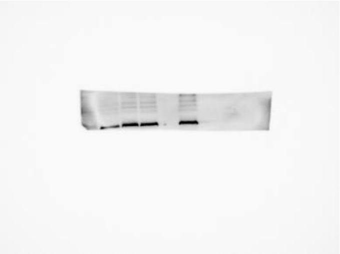
P5	P5	PRMT5 (2)
		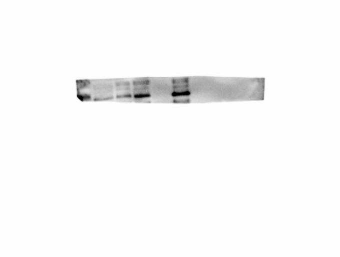
PRMT5	PRMT5 L	PRMT5
tUBULIN	tubulin	TUBULIN2

Fig2		
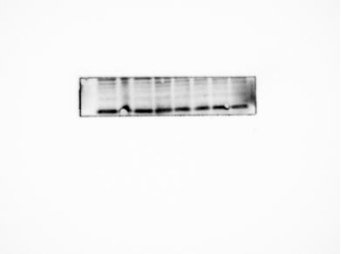	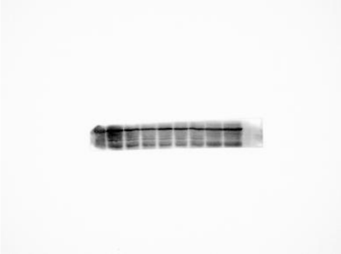	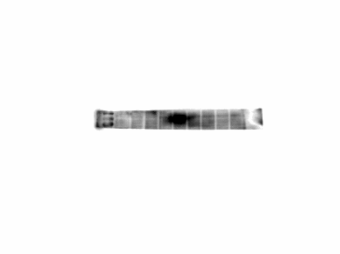
G3BP2		

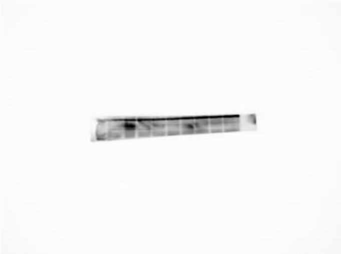	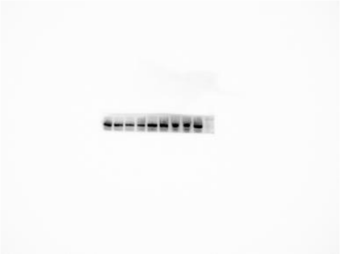	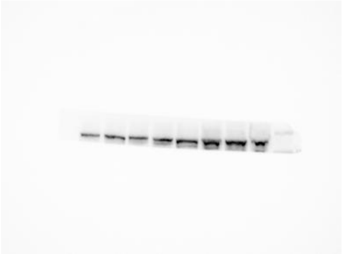
TUBULIN		G3BP2 (2)
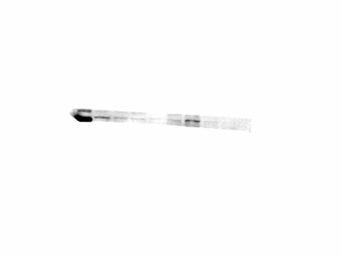	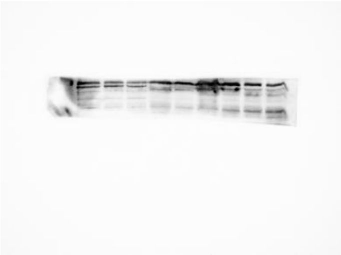	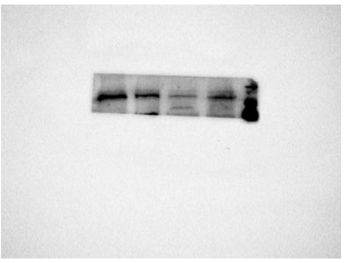
G3BP2 (3)	G3BP2 (4)	G3BP2
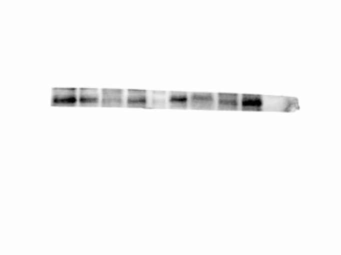	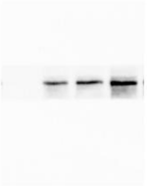	
G3BP2	g3bp2-1 (2)	G3BP2-1
	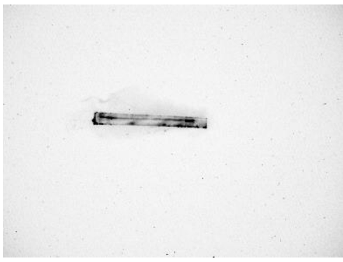	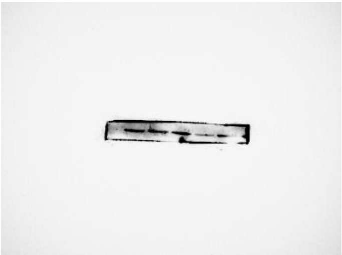
g3bp2-3	G3BP-flag	

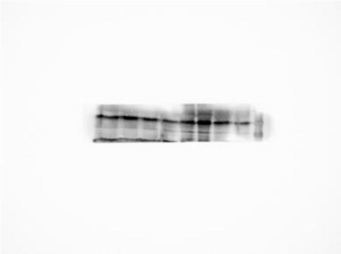	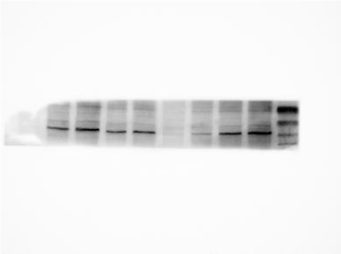	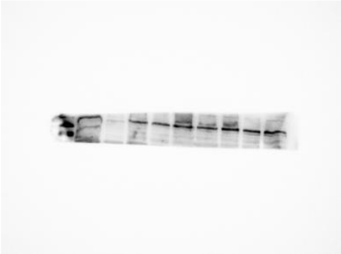
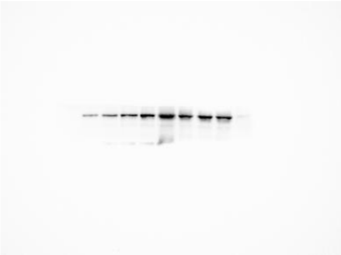		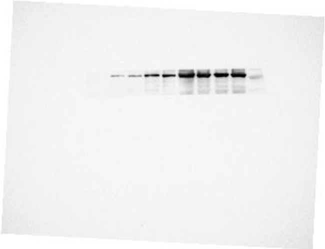
	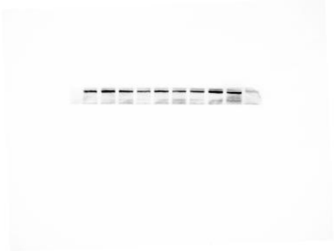	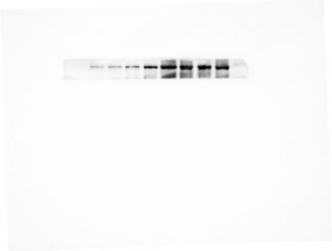
	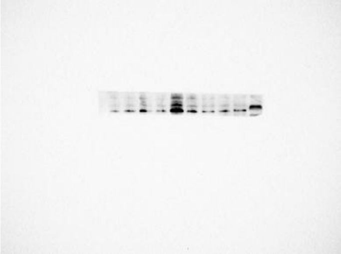	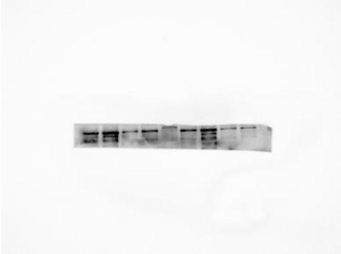
P5 2	P5 10	PRMT5 (2)
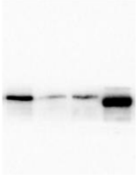	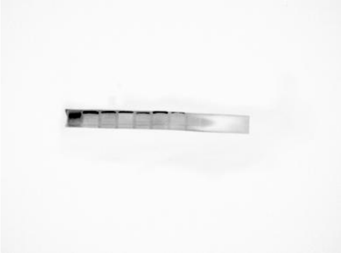	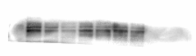

PRMT5 5	PRMT5 5	SUP7
	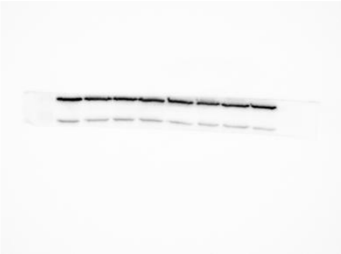	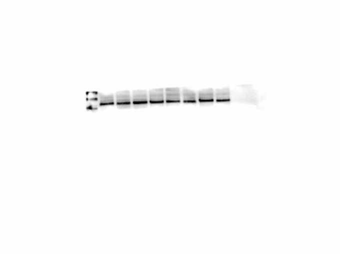
TUB	TUB2	tubulin (2)
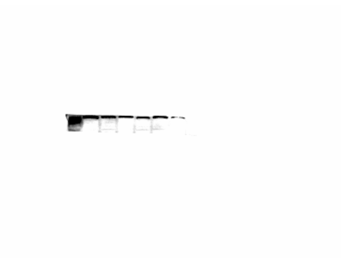	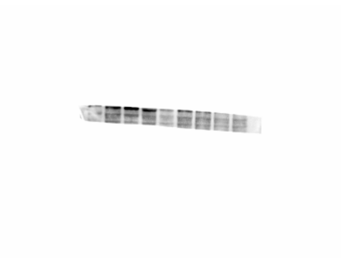	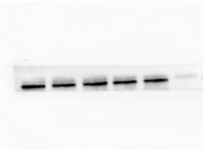
TUBULIN (3)	TUBULIN	tubulin1
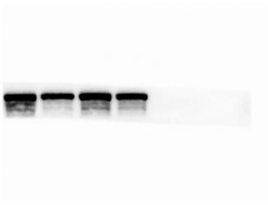	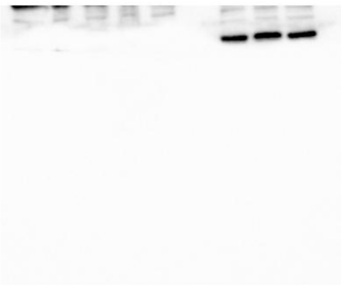	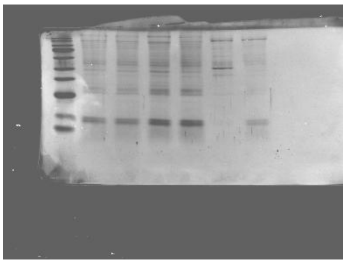
TUBULIN-1	TUBULIN5	UB
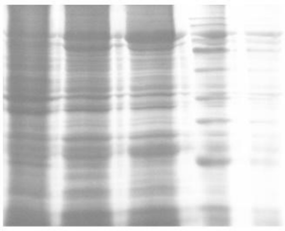	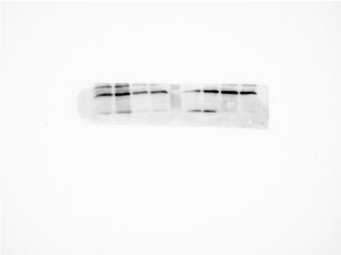	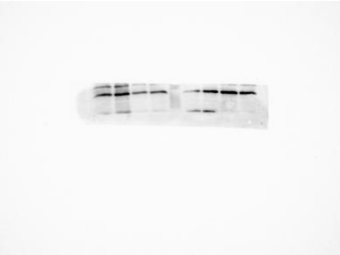
UBIQUTINATION	USP	USP7 (2)

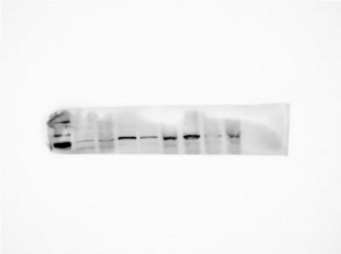	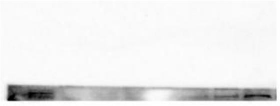	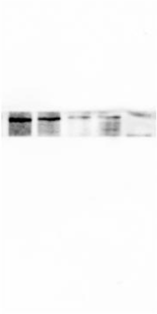
USP7	USP7-3	USP7-4
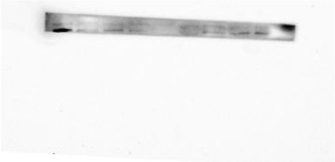	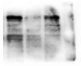	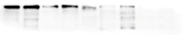

Fig.3		
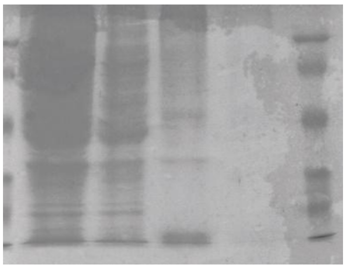		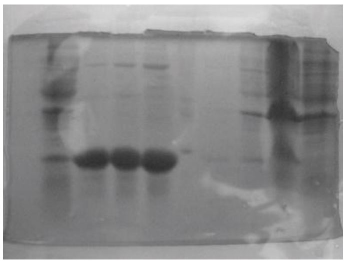

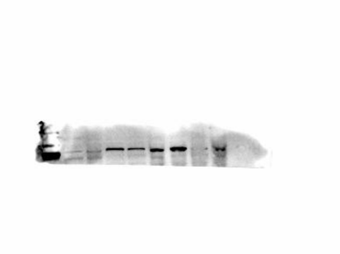	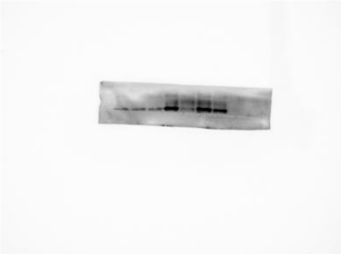	
G3BP2 (2)	G3BP2 (3)	g3bp2
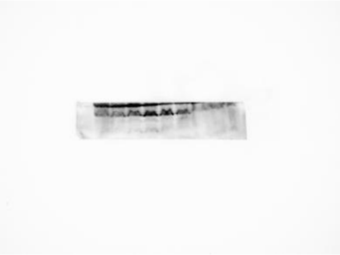		
g3bp2	g3bp2-b	g3bp2-d
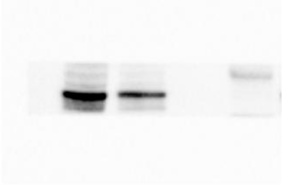	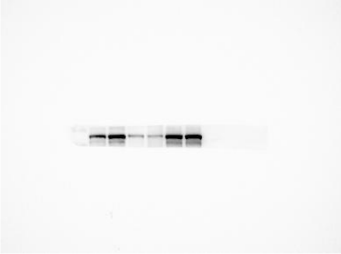	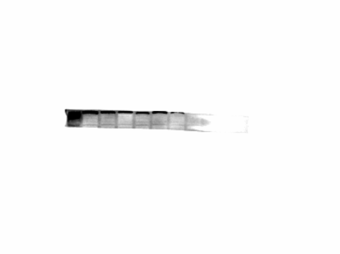
HA-USP7	PRMT5	PRMT5-3
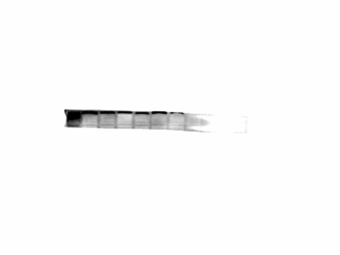	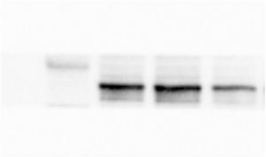	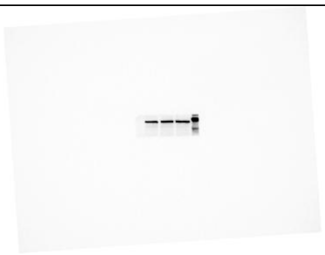
PRMT5-9	prmt5-b	prmt5-d

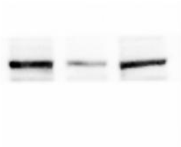	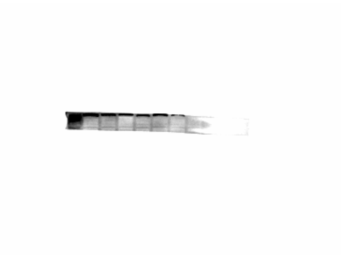	
prmt5-e	PRMT5	tubulin 4
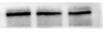	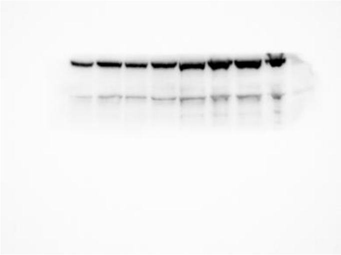	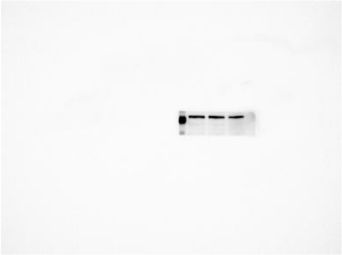
tubulin 5	tubulin	tubulin3
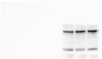	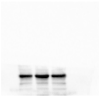	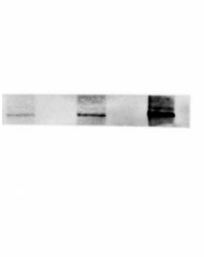
USP	USP	USP6
	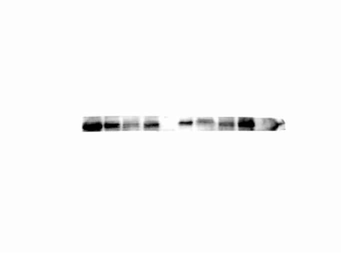	
usp7 (2)	usp7 (3)	usp7 (4)

	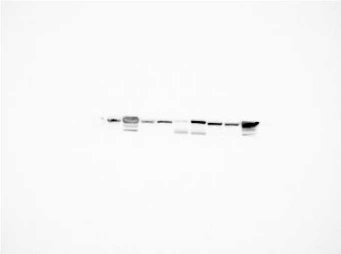	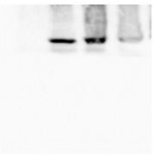
USP7 (5)	USP7 (6)	USP7
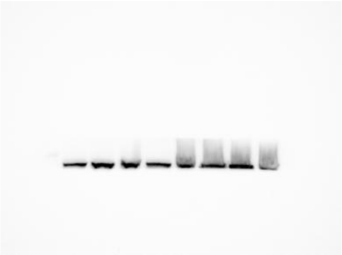		
usp7		

Fig.4		
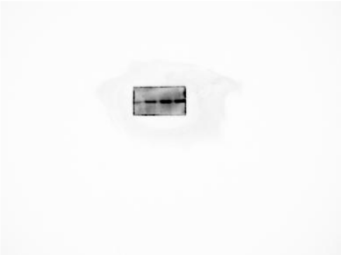	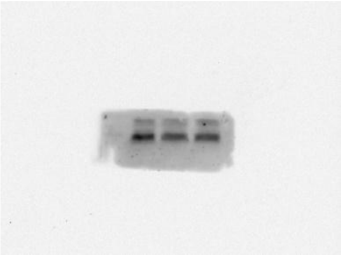	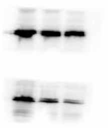
acls3	acly	elovl6
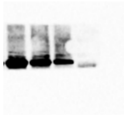	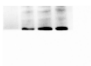	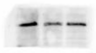

fasn	g3bp2	ppar
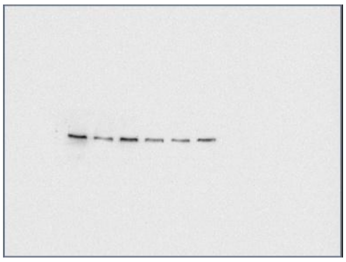	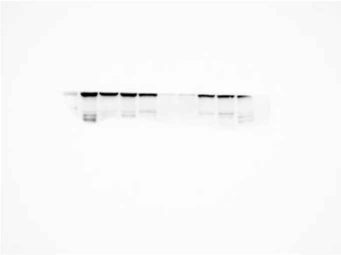	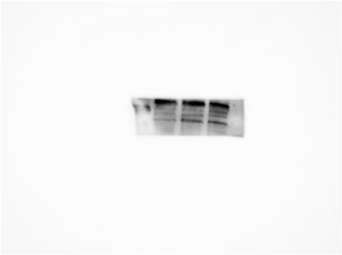
scd1	srebp	tubulin

Fig.6		
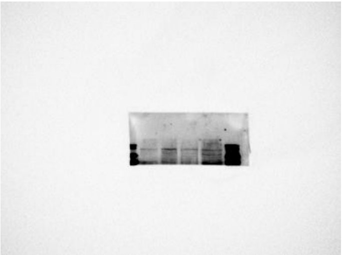	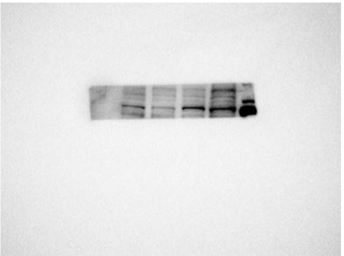	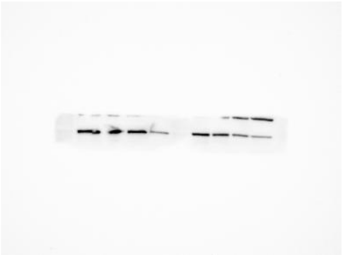
acly	E-cadherin	fasn
	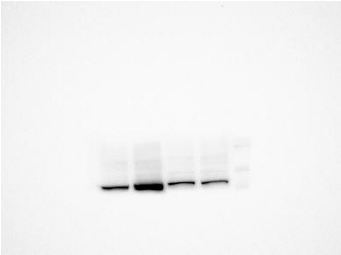	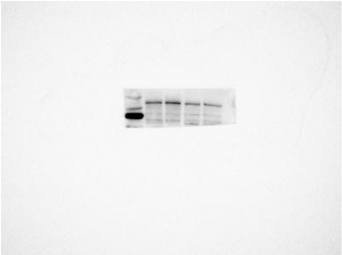
G3BP2	mmp9	Ncadherin

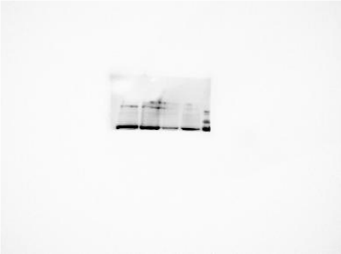	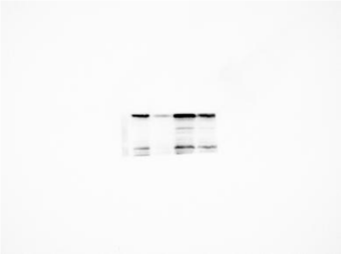	
PRMT5,3	snail	TUBULIN
usp7		

Fig.7		
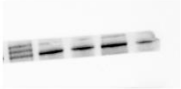		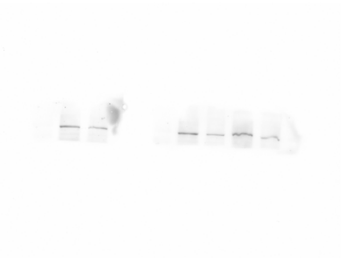
ACLY	ACLY	FASN

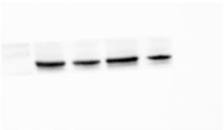		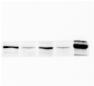
FASN2	G3BP2	G3BP21
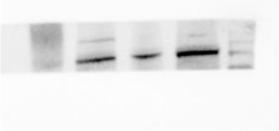	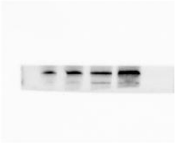	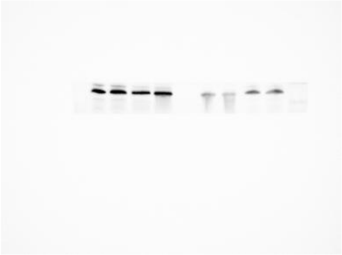
PRMT5	PRMT5	TUBULIN
	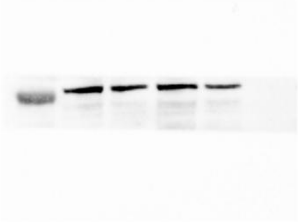	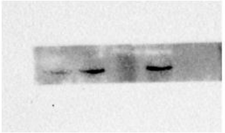
TUBLIN	USP7	Usp7
